# Immunization with a mucosal, post-fusion F/G protein-based polyanhydride nanovaccine protects neonatal calves against BRSV infection

**DOI:** 10.3389/fimmu.2023.1186184

**Published:** 2023-06-09

**Authors:** Teresia W. Maina, Elizabeth A. Grego, Scott Broderick, Randy E. Sacco, Balaji Narasimhan, Jodi L. McGill

**Affiliations:** ^1^ Department of Veterinary Microbiology and Preventive Medicine, Iowa State University, Ames, IA, United States; ^2^ Department of Chemical and Biological Engineering, Iowa State University, Ames, IA, United States; ^3^ Department of Materials Design and Innovation, University at Buffalo, Buffalo, NY, United States; ^4^ Ruminant Diseases and Immunology Research Unit, National Animal Disease Center, Agricultural Research Service, United States Department of Agriculture (USDA), Ames, IA, United States; ^5^ Nanovaccine Institute, Iowa State University, Ames, IA, United States

**Keywords:** human respiratory syncytial virus, bovine respiratory syncytial virus, neonatal calf model, nanoparticles, nanovaccines, immunology, bovine respiratory disease

## Abstract

Human respiratory syncytial virus (HRSV) is a leading cause of death in young children and there are no FDA approved vaccines. Bovine RSV (BRSV) is antigenically similar to HRSV, and the neonatal calf model is useful for evaluation of HRSV vaccines. Here, we determined the efficacy of a polyanhydride-based nanovaccine encapsulating the BRSV post-fusion F and G glycoproteins and CpG, delivered prime-boost *via* heterologous (intranasal/subcutaneous) or homologous (intranasal/intranasal) immunization in the calf model. We compared the performance of the nanovaccine regimens to a modified-live BRSV vaccine, and to non-vaccinated calves. Calves receiving nanovaccine *via* either prime-boost regimen exhibited clinical and virological protection compared to non-vaccinated calves. The heterologous nanovaccine regimen induced both virus-specific cellular immunity and mucosal IgA, and induced similar clinical, virological and pathological protection as the commercial modified-live vaccine. Principal component analysis identified BRSV-specific humoral and cellular responses as important correlates of protection. The BRSV-F/G CpG nanovaccine is a promising candidate vaccine to reduce RSV disease burden in humans and animals.

## Introduction

Human respiratory syncytial virus (HRSV) causes respiratory tract infection and is a constant threat in infants and young children, especially in low- and middle-income countries (LMIC) (1). Global RSV incidences among children 0-5 years cost approximately $33.1 million for new cases. An estimated 100,000 children die annually from RSV, and more than 90% of the infant and child are in LMIC (2, 3). Typical RSV infection causes mild cold-like symptoms but can result in acute lower respiratory tract disease characterized by bronchiolitis or pneumonia in severe cases. Reinfection with RSV is frequent throughout life as RSV-specific neutralizing antibodies induced during natural infection are short lived (4, 5) and infant RSV memory T cell responses fail to prevent reinfection (6). There are no approved RSV vaccines for infants to date and development of a safe efficacious HRSV vaccine is still in progress. Following the recent COVID-19 global pandemic and the relaxation of masking and mitigation strategies, there has been a resurgence of RSV cases globally, with many patients experiencing longer hospitalization periods compared to pre-pandemic standards (7, 8). Thus, the need for efficacious RSV vaccines and therapeutics is more pressing than ever.

To develop effective interventions against HRSV, assorted animal models have been used to evaluate various aspects of virus pathogenesis (9). For RSV research, rodent models have been widely used to provide insights into the basic biology of the infection. However, rodents are less permissive to RSV replication and display significant differences in pathogenesis between viral strains (10). Alternatively, the neonatal calf model presents an opportunity to study a naturally susceptible host-pathogen interaction that resembles RSV infection in humans (reviewed in (11)). Bovine RSV (BRSV) is a major cause of morbidity and mortality in young calves. However, it is also a pathogen associated with bovine respiratory disease (BRD) complex, a multifactorial syndrome in cattle caused by a combination of stress and coinfection with multiple viruses and bacteria (12). HRSV and BRSV are closely related members of the genus *Orthopneumovirus*, in the family *Pneumoviridae*. BRSV and HRSV infection share an age-dependent susceptibility, with disease occurring mostly during fall and winter seasons, and cause comparable pathology of exacerbated respiratory disease and immune responses (13, 14).

Host immune responses induced against RSV are targeted towards major viral transmembrane glycoproteins such as the fusion (F) and attachment (G) proteins located on the surface of the envelope. F protein is required for viral entry and exists in two forms; the post-fusion conformation (15) or the meta-stable pre-fusion conformation (16) with access to varying epitopes (17). F protein is a predominant target for neutralization and protective responses as shown in rodents (18–20), humans (21–23) and cattle (24–27). The highly glycosylated major attachment protein, G, plays a role in viral attachment to host cells but is not required for viral replication (28–31). It is also a target for neutralizing antibodies (32). Candidate vaccines incorporating the G protein have been effective in rodents (32–39), cattle (40–42), and preclinical trials in humans (21, 43). Given their importance in pathogenesis and viral replication, both RSV F and G proteins serve as attractive subunit vaccine candidates. In humans, most RSV subunit vaccine development efforts are focused on the use of a single protein (i.e., the F or G protein) but none have gone beyond clinical trials (44). A multivalent approach, targeting both F and G proteins, may increase the vaccine antigenicity and efficacy by blocking viral fusion and attachment.

Polyanhydrides are biocompatible, biodegradable polymers that have been studied widely for vaccine delivery applications (45, 46) including in cattle (47). Polyanhydride particles are advantageous for their ability to enhance antigen stability, provide sustained antigen release, and induce both antibody- and cell-mediated immunity (48–51). The monomer chemistries that have been commonly used in nanoparticle (NP) formulations are based on sebacic acid (SA), 1,6-bis(*p*-carboxyphenoxy)hexane (CPH), and 1,8-bis(*p*-carboxyphenoxy)-3,6-dioxaoctane (CPTEG). CPH-rich chemistries are relatively more hydrophobic with controlled payload release kinetics occurring on the order of months to years (49, 52) while CPTEG-rich chemistries are relatively more hydrophilic with degradation profiles on the order of days to weeks (52). Manipulation of release rates and hydrophobicity has a direct effect on the elicited immune response with the most hydrophobic formulations eliciting robust humoral and cellular immunity and potentially obviating booster vaccinations (53, 54). Previously, we have reported on the efficacy of a mucosal polyanhydride nanovaccine comprised of 50:50 CPTEG : CPH NPs, encapsulating BRSV post-fusion F and G glycoproteins in the neonatal calf model (27, 55). A single intranasal immunization induced cellular and humoral immunity in the respiratory tract, reduced virus-associated pathology, and decreased the incidence of virus shedding. However, the immunity was partial and failed to induce systemic IgG responses in the face of maternally derived antibodies (MDA). Here, we build on previous work by further optimizing the post-fusion F/G nanovaccine by changing our formulation to 20:80 CPTEG : CPH NPs, encapsulating an adjuvant, CpG oligonucleotide (ODN), and adding a booster dose delivered either intranasally or subcutaneously. We demonstrate that vaccination with BRSV post-fusion F/G CPG nanovaccine is safe and can protect neonatal calves from BRSV infection and elicit cellular and mucosal responses. For the first time we also draw head-to-head comparisons between a subunit nanovaccine and a conventional licensed modified live virus (MLV) vaccine for use in cattle against BRSV. Our findings demonstrate that the protection mediated by the nanovaccine was comparable to the parenteral MLV vaccine.

## Materials and methods

### Polyanhydride monomer, polymer, and NP synthesis

The synthesis of CPTEG and CPH diacids was performed as previously described (56). Melt polycondensation was used to synthesize 20:80 CPTEG : CPH copolymer as described previously (56). Polymer purity, copolymer composition, and molecular weight were evaluated using ^1^H nuclear magnetic resonance spectroscopy (VXR 300 MHz, Varian, Palo Alto, CA).

Before NP synthesis, BRSV post-fusion F protein (GenScript, Piscataway, NJ) and BRSV G protein (KanPro, Lawrence, KS) were separately dialyzed with the use of 10k MWCO Spin-X UF Concentrators (Corning, Corning, NY) and lyophilized for 48 h. A double emulsion nanoprecipitation technique was used to synthesize polyanhydride particles as previously described (57). Briefly, 20:80 CPTEG : CPH copolymer, post-fusion F protein (1.5 wt.%), G protein (1.5 wt.%) and CpG ODN 2007 (1 wt.%) (InvivoGen, San Diego, CA) were dissolved in methylene chloride and sonicated at 30Hz for 30s using a probe sonicator. Following sonication, the solution was quickly poured into a bath of chilled pentane (-4°C) at a solvent to non-solvent ratio of 1:200 and particles were collected by vacuum filtration.

### NP characterization, antigen loading, and release kinetics

Particle size and morphology were determined by scanning electron microscopy (FEI Quanta, FEI, Hillboro, OR). Particle samples were prepared for scanning electron microscopy by dusting tape covered aluminum stubs with dried particles. Samples were then coated with 5nm of iridium using a Cressington 208HR sputter coater (Watford, England, UK). Following coating, samples were analyzed with the use of a FEI Quanta 250 (FEI, Hillsboro, OR). Zeta potential was evaluated by suspending particles in nanopure water and analyzing with a Zeta Sizer Nano-ZS90 (Malvern Panalytical Ltd, Malvern, United Kingdom).

Antigen encapsulation efficiency and protein loading were determined by suspending ~7 mg of particles in 300 µL of 40 mM sodium hydroxide for seven days to accelerate polymer degradation and payload release as described previously (27, 58). Total protein released was quantified using a microBCA protein assay kit (Pierce, Rockford, IL). Release kinetics were performed by suspending ~3 mg of particles in phosphate buffered saline (PBS) and incubated in a 37°C plate shaker for 30 days. Periodically particles were centrifuged and 250 µL of PBS buffer containing released protein was extracted and fresh PBS was added to the incubated particles. A microBCA protein assay kit (Pierce, Rockford, IL) was used to quantify protein in each collected sample. All experiments were performed in triplicate.

Protein stability and antigenicity were evaluated using sodium dodecyl sulphate-polyacrylamide gel electrophoresis (SDS PAGE) and indirect enzyme-linked immunoassay (ELISA).

### Sodium dodecyl sulphate polyacrylamide gel electrophoresis

Post-fusion F protein and G protein encapsulated particles were left to release overnight (18 h) in 300 µL of PBS. Protein structure and stability was analyzed by SDS-PAGE. Each well of a Mini-Protean TGX gel was loaded with 1.5 µg of released proteins. The gel was electrophoresed at 100V for 10 min and then at 140V for 1 h. The gel was incubated in fixative (10% acetic acid and 40% ethanol) for two hours at 4°C then stained overnight with Flamingo fluorescent gel stain (BioRad). The gel was imaged with the use of an iBright™ CL1500 System Imager (ThermoFisher, Waltham, MA).

### Indirect ELISA

Protein stability and antigenicity following release was evaluated by indirect ELISA. High binding 96-well Costar microtiter plates (Corning Life Sciences, Lowell, MA) were coated with 1 µg/mL of released protein and 100 µL of PBS and chilled overnight (4°C). Plates then underwent blocking for two hours at room temperature with 300 µL of 1% bovine serum albumin in PBS with 0.05% Tween 20 (PBS-T). Plates were washed three times with PBS-T and treated with serum from a BRSV infected cow (27) containing post-fusion F protein specific antibody and G protein specific antibody. Plates were incubated overnight at 4°C then washed three times with PBS-T before treatment with an alkaline phosphatase tagged anti-bovine mouse IgG. Following a two-hour incubation at room temperature, plates were washed three times with PBS-T and 100 µL of 1mg/mL phosphate substrate was added to begin the colorimetric reaction. Following 30 min of incubation at room temperature, the optical density of each plate well was read at 405 nm using a Varian Cary 50 Microplate Reader (Varian, Inc., Sunnyvale, CA).

### Calves and vaccination protocol

The experimental procedures were approved by the Iowa State University Institutional Animal Care and Use Committee (protocol 19-202) and the Institutional Biosafety Committee (protocol 19-119).

The study was conducted in two independent replicates with a total of 66, colostrum-replete neonatal, mixed-sex, Holstein calves. All animals used were obtained from the same commercial dairy site located in Eastern Iowa and transported to the Livestock Infectious Disease Isolation Facility at Iowa State University. Enrollment criteria were based upon a satisfactory health assessment upon arrival by a certified veterinarian and colostrum management was confirmed prior to the purchase of the calves. The experiments were conducted indoors in a controlled BSL-2Ag facility consisting of six housing units for the duration of the study. The calves were housed in groups of 4 per pen and each pen was bedded with pine chips and cleaned daily. Each unit was provided with separate equipment for care and sampling. Calves were given starter grain each morning and offered milk replacer twice per day. All calves in the study were provided ad libitum access to water. The animals were blocked by age (7-16 days old). In study one, animals were randomly assigned to three groups (n = 6 animals/group): group 1 received a mucosal prime/systemic boost of saline (control), group 2 received a mucosal prime/mucosal boost of the nanovaccine (In + In BRSV-F/G CpG), and group 3 received a mucosal prime/subcutaneous boost of the nanovaccine (In + Sc BRSV-F/G CpG). In study two, animals were randomly assigned to 4 groups (n = 12 animals/group). Groups 1-3 received the same vaccination regimen as study one. Group 4 received a subcutaneous prime/subcutaneous boost of a commercial vaccine (Elanco Animal Health). The commercial vaccine was a combination modified live virus vaccine (MLV) containing Bovine Viral Diarrhea Virus (BVDV) Type 1and 2, Infectious Bovine Rhinotracheitis (IBR), Parainfluenza Virus 3(PI_3_) and BRSV. Each calf in the nanovaccine group received ~0.5 mg total of recombinant BRSV-F/G proteins and ~290 μg of CpG. Intranasal vaccines were administered in a volume of 5 mL sterile saline, with 2.5 mL injected into each nostril. Subcutaneous vaccines were suspended in 5mL of sterile saline and administered in the right side of the neck. Throughout the studies calves were monitored by a veterinarian for signs of distress or sickness. Two animals were humanely euthanized in study 2 (prior to infection) due to gastrointestinal illness, unrelated to the vaccine protocol or respiratory infection, one from the commercial vaccine (n =11) group and another from the In + Sc BRSV-F/G CpG group (n = 11). The calves exhibited loss of appetite, lethargy, dehydration and fever.

**Figure 1:**
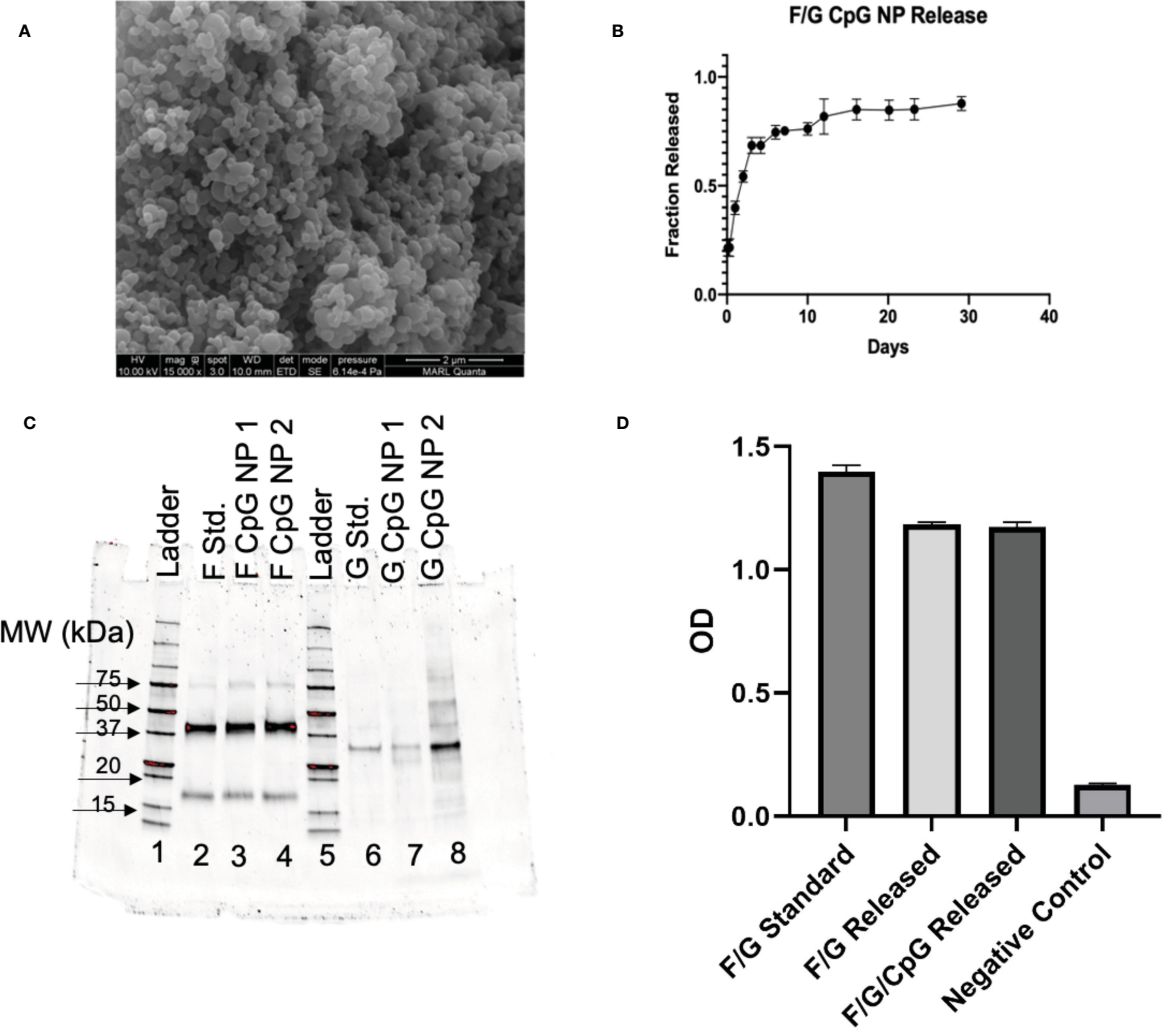
*In Vitro* Characterization of NPs. **(A)** NP size and morphology were determined by scanning electron microscopy. SEM image of 3% F/G CpG 20:80 CPTEG : CPH. NPs showed appropriate morphology and size (188.5 ± 55.8nm). **(B)** Antigen release kinetics were evaluated by suspending ~3 mg of particles in PBS and showed sustained release of protein for 30 days. **(C)** SDS-PAGE analysis was performed on the released proteins to confirm appropriate molecular weight for BRSV antigen released from the particles. **(D)** ELISA plates were coated with 1 µg/mL of released protein. Sera from BRSV-immune cows were diluted 1:1000 and added to the plates. The binding of bovine IgG to the virus or recombinant proteins was measured by absorbance.

The overall experimental design is presented in **Figure 1**. Briefly, pre-immunization (Baseline) blood samples and nasal swabs were collected prior to vaccination. Animals received their first intranasal vaccination at 2-3 weeks of age as in our previous study (27). At four weeks post vaccination, calves received either a mucosal or systemic boost depending on the treatment group. Nasal fluids, serum, and blood were collected every 2 weeks to monitor vaccine-induced immune responses. Three weeks post-boost, all calves were challenged with approximately 10^4^ TCID_50_ of BRSV strain 375 *via* nebulizer on zero days post infection (0 d.p.i.). Calves were monitored daily by a single observer blinded to the treatment groups. The scoring criteria were modified from the University of Wisconsin Calf Health Respiratory Scoring Chart (https://www.vetmed.wisc.edu/fapm/svm-dairy-apps/calf-health-scorer-chs/) and included fever, respiratory rate, and nasal discharge, scored on a scale of 1-3 as described previously (27, 55). The scoring chart also included additional categories for respiratory effort (0 = no effort to 3 = significant effort) and lung auscultation (0 = clear lungs; 1 = wheezing or other harsh lung sounds). Nasal swabs were collected every other day post infection (0, 3, 5, and 7 d.p.i.) to monitor viral shedding by real-time quantitative RT-PCR (RT-qPCR) while blood and serum were collected to monitor BRSV-specific responses. Calves were humanely euthanized on day 7 post-viral infection. In study 1, (n = 6/group) were euthanized by an overdose of pentobarbital (Dolethal, Vétoquinol, France). In study 2, (n =12/group) calves were euthanized *via* intravenous potassium chloride overdose following heavy xylazine/ketamine sedation. A saturated potassium chloride solution was prepared as published by Washington State Animal Care Committee (https://iacuc.wsu.edu/documents/2019/11/sop-6.pdf/) with additional potassium chloride added. At necropsy, whole lungs were removed for physical observations and palpation, quantification of the macroscopic lesions, and bronchoalveolar lavage (BAL) and lung tissue sample collection. Tracheobronchial lymph nodes (TBLN) were collected and analyzed for BRSV specific cellular and humoral immune responses.

### Virus inoculum

BRSV strain 375 was re-isolated from the lung of an infected animal and passaged twice on bovine turbinate cells (BTs), processed through two freeze-thaw cycles, clarified by centrifugation, and stored at −80°C until infection. All calves were challenged with ~10^4^ Tissue Culture Infectious Dose (TCID)_50_ BRSV *via* aerosol delivered by forced-air nebulizer to a mask covering the nose and mouth of the calf. The viral infections were staggered by room and groups of calves were infected every 24 h, with six to twelve calves infected/day. Following infection, room order was followed to eliminate potential cross-contamination between infected and uninfected rooms. The TCID_50_ of the stock virus was determined by titration and confirmed to be free of BVDV and other contaminating viruses (27, 55, 59).

### Pathology evaluation

The extent of gross pneumonic lung consolidation lesions evaluation was performed using a previously described scoring system: 0 = free of lesions; 1 = 1% to 5% affected; 2 = 6% to 15% affected; 3 = 16% to 30% affected; 4 = 31% to 50% affected; and 5 = >50% affected (27, 55, 59). The dorsal and ventral sides of lungs were also photo documented. BAL and lung tissues from affected and unaffected lung tissue were collected for further analysis.

### Sample collection

**Figure 2:**
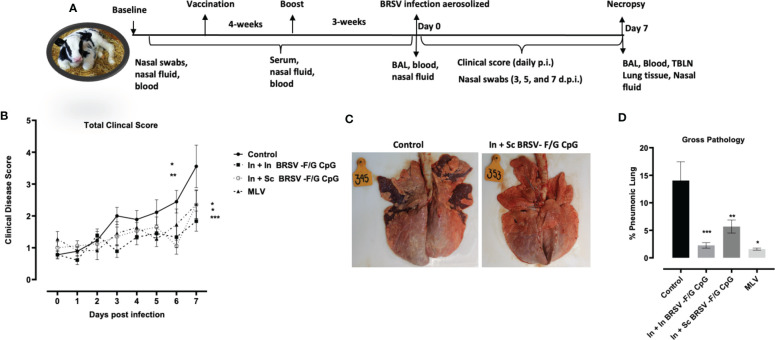
Reduced gross and microscopic pathology in lungs of BRSV-F/G CpG nanovaccine-administered calves. **(A)** Schematic diagram of *in vivo* experiments. **(B)** Calves in all four groups from both studies were monitored daily post infection for fever, respiratory rate, appetite, and nasal discharge and assigned a clinical score. Data represents means ± SEM. Statistical significance determined by 2-way ANOVA with repeated measures, followed by Tukey’s multiple comparisons test**(C)** Representative images from an unvaccinated, control calf and a calf which received an In + Sc BRSV-F/G CpG nanovaccine **(D)** Cumulative gross pathology results from all groups in both studies. Results represent controls (n = 18), In + In BRSV-F/G CpG (n = 18), In + Sc BRSV-F/G CpG (n = 17), and commercial vaccine (n = 11). The graph depicts means ± SEM of each group. *p <0.05, **p<0.01 and ***p<0.001 compared to unvaccinated, control calves as determined by one-way ANOVA with repeated measures, followed by Sidak’s multiple comparisons test.

Blood, nasal swabs, nasal sponges, and BAL were collected as shown in **Figure 2**. Serum was obtained from blood throughout the experiment. For peripheral blood mononuclear cells (PBMCs) isolation, whole blood was collected *via* the jugular vein with a 60 mL syringe containing 2 × acid-citrate-dextrose solution. Cells were isolated from the buffy coat by density centrifugation of blood diluted 1:1 in PBS over Ficoll-Paque (Sigma-Aldrich, USA) and contaminating erythrocytes (RBC) were removed with hypotonic lysis buffer (155 mM NH_4_Cl, 10 mM KHCO_3_) by incubating the cells for 5 minutes. Finally, PBMCs were washed twice in PBS and resuspended in cRPMI culture medium composed of RPMI-1640 (Gibco, Carlsbad, CA) supplemented with 1% antibiotic-antimycotic solution, 1% nonessential amino acids, 2% essential amino acids, 1% sodium pyruvate, 50 μM 2-mercaptoethanol (all from Sigma, St. Louis, MO), and 10% (v/v) fetal bovine sera (FBS) (Sigma-Aldrich, USA).

Nasal swabs were collected for virus quantification from each calf on arrival (Baseline) and 0, 3, 5, and 7 d.p.i. Swabs were stored in serum free minimum essential medium (MEM) and immediately frozen at −80°C until analysis. Nasal fluid samples were collected on arrival, 10 days-post vaccination, pre boost, pre infection (0 d.p.i.) and necropsy (7 d.p.i.) as shown in **Figure 2A**. 1-2-inches of sterile damp sponge was inserted into one nostril of each calf for 5-10 min and placed in a sterile conical tube. The nasal fluids recovered by straining the sponges in a 5mL syringe were then aliquoted and frozen at −80°C until analyzed by ELISA for antibody responses.

BAL was collected at pre infection and necropsy. Pre-infection BAL was collected antemortem as previously described (59). Briefly, calves were lightly sedated using xylazine then intubated using a modified sterile stallion catheter. The collection catheter was inserted in one nostril and passed down to the bronchus and 120 mL of sterile saline was introduced to the lower respiratory tract (LRT) with a catheter-tip syringe, followed by immediate suction to obtain BAL. At postmortem, 500 mL of sterile ice-cold saline was introduced into the lungs through the trachea. After massaging the lungs, the lavage fluid was poured back into sterile collection bottles and placed on ice. Cells from BAL were filtered, and any contaminating RBC were lysed with hypotonic lysis buffer. Fresh BAL samples were submitted to the Iowa State University Veterinary Diagnostic Laboratory (VDL) for cytospins smear slide preparation and differential staining (Modified Wrights Stain). For BRSV specific responses, only postmortem BAL samples from study 1 were used due to technical challenges encountered while acquiring BAL from calves euthanized with an overdose of potassium chloride in study 2. TBLN were also collected postmortem, and the cells obtained by mechanical dissociation of the tissue were suspended in cRPMI. Cell suspensions were then filtered through 40 μm cell strainer, washed, counted, and resuspended in cRPMI.

### Lung tissue histological scores

Microscopic analysis was performed on formalin-fixed lung, trachea and TBLN tissue samples by a blinded, veterinary pathologist at the ISU Veterinary Diagnostic Lab, Ames, IA. Cell infiltration and the extent of the lung section affected were graded based on the degree and type (lymphocytic, neutrophilic, macrophage) of cellular infiltrate: – (absent) no infiltrating cells detected; (+) (insignificant) few scattered solitary cells detected; + (mild) small areas with infiltration of few cells; ++ (moderate) multifocal areas with evident infiltration; or +++ (severe) widespread areas with prominent infiltration.

### BAL cytology

Differential cell counts were performed by a blinded pathologist on BAL samples and a minimum of 500 cells were counted per sample, excluding epithelial cells. The results of the differential cell counts were expressed as a percentage composition.

### Serology and cytokine measurements or ELISA

Cytokine secretion was determined in the stored supernatants from PBMCs and TBLNs using commercial enzyme-linked immunosorbent assay (ELISA) kits for bovine interleukin 17A (IL-17) and interferon gamma (IFNγ) (Kingfisher Biotech, Inc, USA) in accordance with the manufacturer’s instructions.

In serum, total BRSV-specific bovine IgG_1_ was quantified using the commercial Svanovir BRSV Ab kit (Svanova, Boehringer Ingelheim), according to the instructions provided by the manufacturer.

Nasal fluid IgA and serum total IgG specific to post-fusion F or G were quantified with indirect ELISAs. Total BRSV-specific bovine IgA was quantified by coating 96-well ELISA plates overnight at 4°C with 100 µl/well of BRSV stock (~10^4^ TCID_50_). Negative control wells were coated with 100 µl/well cell culture media prepared from uninfected BT cells. Post-fusion F or G-specific antibodies were quantified by coating 96-well ELISA plates overnight at 4°C with 3 μg/mL of either post-fusion F protein or G recombinant protein. Plates were blocked with 4% BSA in PBS. Nasal fluid samples diluted 1:4 and plated in duplicates. Serum samples were diluted 1:100 and plated in duplicates. Sheep anti-bovine IgA-HRP (Bethyl Laboratories) was used at 0.5 µg/mL for detection. Rabbit anti-bovine total IgG-HRP (Bethyl Laboratories) was used a 0.5 µg/mL for detection. Pierce 1-Step Ultra TMB Substrate (ThermoScientific Pierce) was used to develop the plates. Data values were calculated by subtracting absorbance values at 450 nm of wells containing control uninfected BT supernatant from wells containing BRSV antigen. Data was expressed as relative ELISA values by dividing with pooled baseline samples.

### Serum virus neutralization assays

Serum samples collected at baseline, pre infection (0 d.p.i.) and necropsy (7 d.p.i.) were submitted to the Iowa State University Veterinary Diagnostic Laboratory (Ames, Iowa) for evaluation of BRSV-specific neutralization titers. The cut-off for the assay is 1:4.

### Antigen recall assays

PBMCs and TBLN cells were resuspended in 1 ml of PBS containing 10 μM of the Cell Trace Violet (CTV) stain and incubated for 20 min at 37 °C. CTV labeling was quenched by adding four volumes of RPMI, and washed twice with RPMI (Invitrogen, Life Technologies) before stimulating the cells for antigen recall assay. 5x10^6^cells/mL PBMCs and TBLN cells were added to U-bottom 96-well plates (Corning, NY, USA) in duplicates. Cells were stimulated with culture medium as a negative control, 5 μg/mL Concanavalin A (ConA) as a positive control, 5 μg/mL post-fusion F protein, 5 μg/mL G protein or BRSV for 5 days (PBMCs and TBLN cells) at 37°C and 5% CO_2_. After incubation, supernatants were collected and stored for cytokine ELISA at −80°C. Simultaneously, PBMCs and TBLN cells were immediately surface stained for T cell markers. Briefly, cells were suspended in flow cytometry staining (FACS) buffer (10% FBS and 0.02% NA-azide in PBS) and stained for 30 min in the dark at 4°C with 10 μg/mL of mouse anti-bovine CD4-FITC (clone CC8) and CD8α-Alexa647 fluorochrome-conjugated monoclonal antibodies (Bio-Rad Laboratories, USA). Viability of the cells after the 5-day incubation was also measured using the LIVE/DEAD™ Fixable Near-IR Dead Cell Stain Kit (ThermoFisher Scientific, USA) according to manufactures instructions. After washing, cells were fixed with BD FACS lysis buffer (BD Biosciences, USA) for 10 min at room temperature (RT), washed, and resuspended in FACS buffer. Cells were acquired using a BD FACS Canto II (BD Biosciences, USA) and analyzed with FlowJo Software (Tree Star Inc., San Carlos, CA, USA). Percentages of cell proliferation were expressed over mock treated cells.

### Quantification of virus replication/viral burden

BRSV non-structural 2 (NS2) gene was used to quantify viral shedding in nasal swabs and viral burden of snap frozen lung tissues by q-PCR as previously described (27, 55, 59). Trizol Reagent (Invitrogen, Life Technologies) was used to isolate total RNA from two representative lung tissue samples, gross-lesioned and non-lesioned stored in RNAlater (Invitrogen, Life Technologies) and frozen nasal swabs stored in media. Thawed swabs were vigorously vortexed, and the supernatant was used for qPCR. For the reaction, Taqman RNA-to-CT 1-step kit (Applied Biosystems) was used and DNA sequences coding for bovine RPS9, and BRSV NS2, both cloned separately into PCR2.1-TOPO vectors were used as templates for the standard curve construction. Each reaction was run in triplicate and standard curves for both genes were run in parallel with test samples. Viral NS2 copy numbers were calculated using the standard curves and normalized to RPS9 to correct for differences in sample input. qPCR was run on a ThermoFisher Scientific QuantStudio 3 Real-Time PCR machine. Primer and probe sequences have been previously published (27, 55, 59).

### Statistical analyses

All statistical analyses were performed in GraphPad Prism 9.4.1 (GraphPad Software, Inc, San Diego, CA, USA). Results were tested for normal distribution prior to data analysis. Data were graphed and expressed as arithmetic mean ± standard error of the mean (SEM). Statistical significance was determined by one-way Analysis of Variance (ANOVA) or two-way ANOVA with repeated measures followed by Sidak’s, Tukey’s or Dunnett’s multiple comparisons test where appropriate. While single measurement data were analyzed by unpaired t-test or Mann-Whitney test, as appropriate. For relative gene expression analyses, ΔΔCt values were used to calculate 2^-ΔΔCt^. Differences with p ≤ 0.05 were considered significant and 0.05<p ≤ 0.1 were considered a trend.

### Principal component analysis

PCA was used for two different purposes: the parameterization of data and then correlative analysis. The data contain fifteen different measurement categories, with the data spanning total clinical score, gross lung pathology, lung histopathology score, BAL cytology, nasal virus shedding and lung viral burden. Across all of these categories, the complete available data was analyzed. The ability to simultaneously analyze all of the data to capture all correlations is a primary benefit of using PCA. That is, relationships in the data are hard to visualize and PCA is a method to compress the data. The challenge is that each of the categories had different numbers of measures and therefore integrating into a single analysis is challenging.

At the onset, the data categories had different dimensionalities and different scales, levels of uncertainty, and standard deviations. That is, some of the categories contain multiple variables while others contain a single variable. Therefore, the data cannot be combined in one matrix as is because those categories with more variables are prone to have a larger impact on the model. Additionally, within categories, it is likely that some of the variables are correlated with each other, while the analysis should have the different variables as uncorrelated as possible. To address these challenges, PCA was used in a unique manner whereby PCA was first applied to each of the data categories individually to convert the data to one dimension with a common format, while maintaining the majority of information in the original data. This maintained the measures of the same dimensionality and potential statistical relevance, while also minimizing our bias. Three of the categories were one dimensional and were kept the same. PCA was applied to the other 12 categories. The component for each of these 12 categories is a linear combination of all of the input measurements, with the linear combination accounting for the inter-correlations in the data so that all of the information is maintained but provided in a unified manner. In this way, each component can be considered as an equation which captures the relationships in the data in a low dimensional manner. Therefore, this first stage consisted of 12 analyses, resulting in a suitable input for the larger analytical component. To ensure that the results were not overly skewed by limiting each measure to one dimension, the analysis was repeated using a different number of dimensions and ensuring that the results were consistent. This extra testing found consistent results and therefore the analysis is sufficiently robust.

The informatics developed data was then analyzed in a standard PCA approach. The data was converted into two matrices: the scores matrix which described the relationship between the different cattle, while the loadings matrix defined the correlations between the different descriptors and responses. In this way, the loadings mathematically defines the new axis space, while the scores is then the plotting of the original data in the new axis system. The relationship between the different treatment groups, as well as which aspects of the responses caused the differences, were captured in the analysis. In this way, the unique integration of 13 different PC analyses on the data of various dimensionalities and time scales resulted in an interpretable visualization of the data and a single correlative value.

## Results

### NP characterization and *in vitro* antigenicity

Polymer purity and molecular weight were evaluated with (1)H nuclear magnetic resonance (NMR) spectroscopy (VXR 300 MHz, Varian, Palo Alto, CA). The copolymer composition was 15% CPTEG and 85% CPH. The polymer had an average molecular weight of 9,063 g/mol with a polydispersity index (PDI) of 2.94. Particle morphology and size was analyzed using scanning electron microscopy (SEM, **Figure 1A**). NP morphology was appropriate and consistent with previous work (60) and the particles were 189 ± 56 nm in size. The zeta potential of the particles was 6.18 ± 0.28 mV.

Through base extraction and utilization of a microBCA protein assay kit, the antigen encapsulation efficiency was determined to be 72.5%, leading to a total protein loading of 2.1 wt.%. Antigen release kinetics were measured for one month. The NPs exhibited an initial burst release of protein within the first week, followed by a sustained release of protein for three weeks (**Figure 1B**). Overall, the NPs released 88.8% of the total protein payload in 30 days (**Figure 1B**).

Structure stability and appropriate folding of post-fusion F and G following particle release were analyzed by SDS-PAGE. Based on protein standards, stable, intact BRSV antigen (characterized by bands at ~40 kDa and ~17 kDa) was released from the NPs with minimal degradation (**Figure 1C**). Antigenicity of released protein was analyzed by ELISA (**Figure 1D**). Wells coated with protein release samples had an optical density (OD) similar to wells coated with protein alone, indicating successful antibody binding to released protein and preservation of antigenicity, as reported previously (27).

### Prime-boost immunization with BRSV-F/G CpG nanovaccine induced protection from clinical disease and lung pathology following BRSV challenge in neonatal calves

Following challenge, calves were monitored daily for clinical signs including rectal temperature, nasal discharge, cough, and respiratory effort from experimental day 0 (0 days post infection (d.p.i.)) to 7 d.p.i. No calves had elevated clinical signs on 0 d.p.i. All control calves developed clinical signs, varying from mild to severe starting from 3 d.p.i. and peaking at 5-7 d.p.i. In contrast, clinical signs exhibited by nanovaccine immunized calves were absent to mild throughout the study. Calves receiving the In + In BRSV-F/G CpG nanovaccine and calves receiving the In + Sc BRSV-F/G CpG nanovaccine had lower total clinical scores on 6 d.p.i. (*p<0.05 and **p<0.01 respectively) and 7 d.p.i. (***p< 0.001 and *p<0.05 respectively) compared to the non-vaccinated control group (**Figure 2B**). In contrast, no differences were observed in clinical disease scores between MLV immunized calves and those receiving the nanovaccines (**Figure 2B**). Overall, the calves that received the BRSV-F/G CpG nanovaccine were clinically protected and we observed no signs of vaccine enhanced disease (VED).

Necropsy was performed on 7 d.p.i. and the lungs were evaluated macroscopically for gross pathology as previously described (27, 55, 59). Representative gross lung images are depicted in **Figure 2C**, and the percentages of grossly affected pneumonic lung are shown in **Figure 2D**. Following viral challenge, the non-vaccinated calves (i.e., controls) had more severe lung lesions typical of bronchointerstitial pneumonia. The In + In BRSV-F/G CpG, In + Sc BRSV-F/G CpG, and MLV vaccinated calves exhibited significantly less lung pathology (***p< 0.001, **p<0.01, *p<0.05 respectively) compared to the non-vaccinated group (**Figure 2D**). No differences in lung pathology were observed between calves receiving the MLV or nanovaccine regimens.

**Figure 3:**
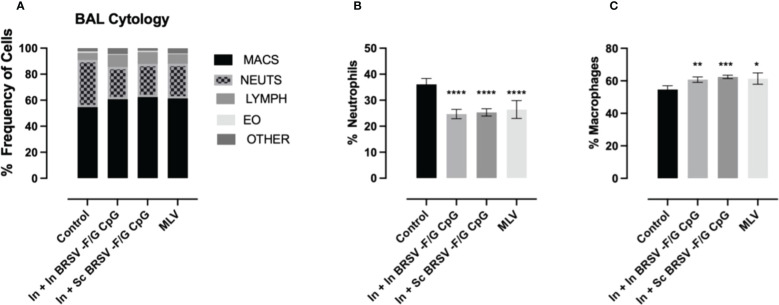
Reduced neutrophil infiltration in BAL of BRSV-F/G CpG nanovaccine-administered calves following BRSV challenge. Differential cell counts in BAL fluid of neonatal calves challenged with BRSV was performed by a blinded clinical pathologist on 7 d.p.i. **(A)** Proportion of granulocytes in BAL evaluated by cytology. Differences in the relative proportions of neutrophil **(B)** and macrophages **(C)**. The results are expressed as a percentage composition. The graphs represent controls (n = 18), In + in BRSV-F/G CpG (n = 18), In + Sc BRSV-F/G CpG (n = 17), MLV (n = 11) treatment groups. The graph depicts means ± SEM of each group. Statistical significance was determined by two-way ANOVA with Tukey’s post test. *p<0.05, **p<0.01, ***p<0.001, ***p<0.001 compared to unvaccinated control calves.

Cytology was performed on fresh BAL obtained at necropsy and differentially stained to evaluate relative frequency of different immune cells. Severe cases of BRSV in neonatal calves are characterized by significant airway neutrophil infiltration at the site of infection (9, 61). Alveolar macrophages were the predominant cell type identified in the BAL samples with approximately 60% frequency, followed by neutrophils between 26-36%, lymphocytes between 7-10%, eosinophils between 0.4-1% and other cells between 1-8% (**Figure 3A**). We observed higher proportions of neutrophils in the BAL of non-vaccinated control animals compared to calves that received the nanovaccine formulations by either homologous or heterologous route of administration (****p<0.0001) or the MLV (****p<0.0001) (**Figure 3B**). We also observed relatively lower frequency of macrophages in our non-vaccinated controls compared to the calves that received the In + In BRSV-F/G CpG and In + Sc BRSV-F/G CpG nanovaccine (**p<0.01, ***p<0.001 respectively) and MLV (*p<0.05) (**Figure 3C**). No differences were observed in the proportions of BAL lymphocytes, eosinophils, or other cells between groups.

### Immunization with the BRSV-F/G CpG nanovaccine induced viral protection in the lungs of neonatal calves following BRSV challenge

**Figure 4:**
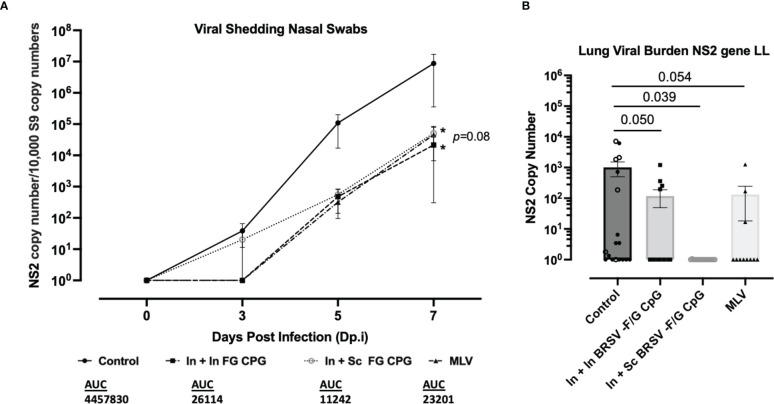
Nanovaccine induced viral protection in the lower and upper respiratory tract of neonatal calves. **(A)** Nasal swabs were collected on 0, 3, 5, and 7 d.p.i. to evaluate virus shedding. BRSV RNA was detected by RT-qPCR, and viral NS2 copy numbers were calculated using standard curves. The graphs include Controls (n = 18), In + In BRSV-F/G CpG (n = 18), In + Sc BRSV-F/G CpG (n = 17), MLV (n =11) and depict means ± SEM of each group. Statistical significance was determined by two-way ANOVA with a Tukey *post hoc* test **(B)** After euthanasia on 7 d.p.i. lung tissue samples were collected from 2-3 representative lesion and non-lesion sites of the lungs. Lung tissues were normalized to the housekeeping gene, RPS9, to correct for differences in input material. Statistical significance was determined by one-way ANOVA with a Holm-Šídák *post hoc* test. AUC, Area under curve.

Next, we assessed the effect of prime-boost vaccination with the BRSV-F/G CpG nanovaccine on viral clearance. Following BRSV challenge, all calves were sampled for nasal swabs at days 0, 3, 5, and 7 post infection to track viral shedding by RT-PCR. Viral RNA was not detected in any groups on 0 d.p.i. Viral RNA was detectable by 3 d.p.i. but decreased in the BRSV-F/G CpG nanovaccine- immunized and MLV immunized calves compared to the nonvaccinated controls. By 7 d.p.i., less viral RNA was detected in the upper respiratory tract (URT) of calves that received the BRSV-F/G CpG nanovaccines (both *p<0.05) and MLV (*p=*0.080) compared to non-vaccinated calves (**Figure 4A**). Quantitative differences in viral shedding between groups were estimated by calculating the area under curve (AUC). Within the 7 days following challenge, the non-vaccinated control calves shed the most virus (4,457,830 AUC), In + Sc BRSV-F/G CpG and MLV shed comparable amounts of virus (26,114 and 23,201 AUC respectively), and the In + In BRSV-F/G CpG shed the least amount of virus (11,242 AUC) (**Figure 4A**). Consistent with the nasal virus shedding data, qPCR analyses of lung tissue on 7 d.p.i. revealed significantly less viral RNA in the In + Sc BRSV-F/G CpG vaccinated calves (*p<0.05) and a marked (and trending) decrease in the In + In BRSV-F/G CpG (*p=*0.050) and MLV (*p=*0.054) calves compared to non-vaccinated controls (**Figure 4B**). Viral shedding and viral burden results indicate that immunization with the BRSV-F/G CpG nanovaccines reduced the viral load in both the LRT and URT of neonatal calves with BRSV infection (**Figures 4A, B**).

### Effects of the BRSV Post-F/G CpG nanovaccine on humoral immune responses

We next evaluated serum F, G and BRSV-specific IgG responses and virus neutralizing antibody titers. All calves were colostrum replete and had BRSV-specific MDA at the time of enrollment. BRSV-specific antibody titers continued to decline throughout the experiment. We observed no differences in whole virus IgG_1_ responses (**Table 1**), in the post-F- or G- specific total IgG serum responses (**Table 1**), or neutralizing antibody titers (**Table 2**) between groups, regardless of the vaccination regimen.

**Table 1:** BRSV-specific IgG responses.

	Days post infection	Control	In + In BRSV - F/G CpG	In + Sc BRSV - F/G CpG	MLV
**BRSV specific**	**0**	0.68 (0.1)	0.84 (0.08)	0.92 (0.06)	0.79 (0.06)
	**7**	1.04 (0.04)	1.05 (0.06)	1.07 (0.06)	0.98 (0.06)
**Post-F specific**	**0**	1.2 (0.15)	1.3 (0.13)	1.4 (0.11)	1.5 (0.19)
	**7**	1.3 (0.15)	1.3 (0.1)	1.5 (0.16)	1.2 (0.15)
**G specific**	**0**	0.65 (0.09)	0.65 (0.08)	0.87 (0.01)	0.81 (0.13)
	**7**	0.6 (0.07)	0.07 (0.07)	0.86 (0.09)	0.51 (0.06)

Serum absorbance values at 450 nm associated with anti-bovine respiratory syncytial virus (BRSV) IgG_1_; or anti-BRSV post-F or G total IgG in control and vaccinated calves at 0 and 7 d.p.i. Antibody responses were detected by ELISA. ELISA units are reported as mean (SEM). Controls (n = 18), In + In BRSV-F/G CpG (n = 18), In + Sc BRSV-F/G CpG (n = 17), MLV (n = 11).

**Table 2:** BRSV neutralizing antibodies.

	Control	In + In BRSV- F/G CpG	In + Sc BRSV- F/G CpG	MLV
**Baseline**	157 (20.9)	174 (18.5)	175 (20.2)	197 (21.5)
**0 d.p.i.**	88 (10.6)	106 (15.6)	96 (13.6)	113 (11)
**7 d.p.i.**	112 (19.9)	111 (18.8)	117 (21.2)	112 (25.8)

BRSV neutralizing antibody titers were measured in serum at baseline (pre-immunization), 0 d.p.i. (prior to challenge) and 7 d.p.i. Statistical significance was determined by two-way ANOVA followed by Tukey’s multiple comparisons test, controls (n = 18), In + In BRSV-F/G CpG (n = 18), In + Sc BRSV-F/G CpG (n = 17), MLV (n = 11). Data presented as mean (SEM).

We next examined BRSV-, post-F-, and G-specific IgA production in the respiratory tract. We observed no differences in IgA responses in the nasal fluid between control and vaccinated groups on either day 0 (pre-infection) or day 7 post-challenge (**Table 3**). Similarly, we observed no differences between the control group and vaccinated groups in virus-specific IgA concentrations in BAL fluid collected prior to infection. However, in study 1, we observed greater BRSV-specific IgA responses in the LRT from BAL collected from the In + Sc BRSV-F/G CpG vaccinated group (*p=*0.053) compared to the unvaccinated controls on 7 d.p.i. (**Table 3**). 7 d.p.i BAL samples from study 2 calves were excluded from analysis due to technical challenges.

**Table 3:** Prime-boost administration of BRSV-F/G CpG nanovaccine enhanced BRSV specific mucosal IgA responses in neonatal calves following BRSV challenge.

Nasal fluid	Days post infection	Control	In + In BRSV -F/G CpG	In + Sc BRSV -F/G CpG	MLV
BRSV specific	**0**	7.72 (1.03)	8.6 (0.99)	7.93 (0.78)	9.50 (0.74)
	**7**	7.31 (1.07)	8.99 (0.85)	9.04 (0.93)	10.28 (1.15)
Post-F specific	**0**	7.42 (1.53)	9.72 (1.96)	10.10 (2.30)	4.56 (0.96)
	**7**	8.98 (2.13)	8.23 (1.85)	8.65 (1.99)	4.37 (0.88)
G specific	**0**	21.83 (2.36)	27.53 (2.37)	25.94 (2.47)	23.27 (2.86)
	**7**	23.58 (3.02)	25.62 (2.48	23.27 (2.90)	26.66 (3.72)
BAL fluid					
BRSV specific	**0**	1.23 (0.42)	0.85 (0.08)	1.42 (0.43)	ND
	**7**	1.66 (0.22)^a^	2.91 (0.57)	2.38 (0.30)^b^	

^a,b^ Within a row, means with unlike superscripts differ 0.05 < P 0.10.

IgA relative ELISA units for nasal fluid collected on 0 and 7 d.p.i. specific for whole BRSV, post-F protein or G protein determined by indirect ELISA. Results represent controls (n = 18), In + In BRSV-F/G CpG (n = 18), In + Sc BRSV-F/G CpG (n = 17), commercial vaccine (n = 11). IgA titers of BAL collected at 0 and 7 d.p.i. during study 1 (n = 6), specific for whole BRSV determined by indirect ELISA. Data represent means ± SEM.

### BRSV-F/G CpG nanovaccine induced greater BRSV specific cellular immune responses following BRSV challenge in neonatal calves

To assess the BRSV-F/G CpG nanovaccine effect on cellular immunity, we analyzed antigen-specific CD4^+^ and CD8^+^ T cell responses in peripheral blood and TBLN using *ex vivo* antigen recall assays. Cells were stimulated for five days with whole BRSV, post-F antigen, or G antigens to measure T cell proliferation by dilution of CTV by flow cytometry. Cell culture supernatants were analyzed for secretion of IFNγ and IL-17 cytokines by ELISA. We observed no antigen specific CD4^+^ or CD8^+^ T cell proliferative responses by PBMCs prior to challenge, regardless of treatment group (Data not shown).

**Figure 5:**
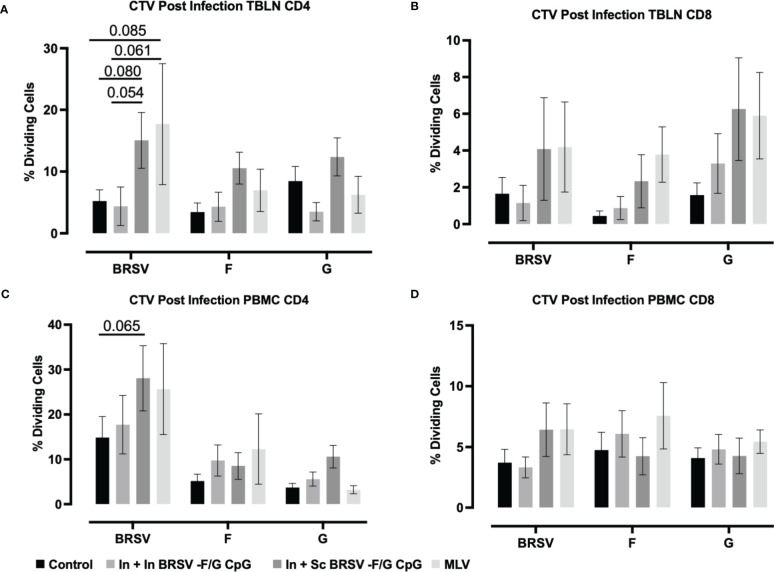
BRSV-specific T cell responses in peripheral blood and TBLN of BRSV-F/G nanovaccine-administered calves. Calves were experimentally infected as described in **Figure 2A**. TBLN and PBMCs was collected at 7 d.p.i. and stimulated either with culture medium, 5 μg/mL ConA, 5 μg/mL post-F protein, 5 μg/mL G protein or BRSV for 5 days. After incubation, PBMCs and TBLN were surface stained for T cells markers and proliferation of T cells detected by flow cytometry. **(A, B)** Frequency of CD4 and CD8 dividing cells specific to BRSV, post-F protein, or G protein in TBLN collected on 7 d.p.i. **(C, D)** Frequency of CD4 and CD8 dividing cells specific to BRSV, post-F protein, or G protein in PBMCs collected on 7 d.p.i. Data is presented as percentages of cell proliferation expressed over mock treated cells. Data represent means ± SEM. Statistical significance was determined by two-way ANOVA with Tukey’s post test. Controls (n = 18), In + in BRSV-F/G CpG (n = 18), In + Sc BRSV-F/G CpG (n = 17), commercial vaccine (n = 11).

In TBLN we observed an increased frequency of BRSV-specific CD4^+^ T cell responses in calves that received the subcutaneous nanovaccine boost compared to non-vaccinated control calves (p=0.080) and compared to calves receiving the homologous intranasal nanovaccine (p = 0.054); however, we observed no differences between control and In In BRSV-F/G CpG calves in the TBLN responses (**Figure 5A**). Calves that received the MLV vaccine had a tendency for increased CD4^+^ T cell proliferative responses compared to both the control (*p=*0.085) and In + In BRSV-F/G CpG (*p=*0.061) calves, but there were no differences between the In + Sc BRSV-F/G CpG and the MLV calves. We observed no differences in antigen specific CD8^+^ T cell proliferation in TBLN samples following BRSV challenge (**Figure 5B**). Following challenge, we noted greater virus-specific CD4^+^ T cell proliferative responses in PBMCs of calves that received the In + Sc BRSV-F/G CpG nanovaccine in response to BRSV restimulation (*p=*0.065) compared to the unvaccinated controls (**Figure 5C**). No differences in antigen specific CD8^+^ T cell proliferative responses were noted after infection (**Figure 5D**).

**Figure 6:**
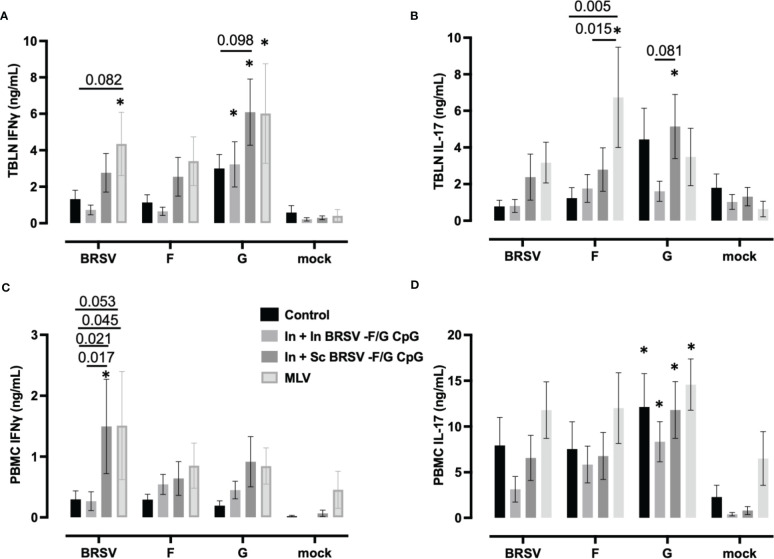
Cytokine responses in the peripheral blood and tracheobronchial lymph node (TBLN) of BRSV-F/G nanovaccine-administered calves. TBLN cells **(A, B)** and PBMCs **(C, D)** were collected at necropsy and stimulated for 5 days either with culture medium as a negative control, 5 μg/mL Concanavalin A (ConA) as a positive control, 5 μg/mL post-F protein, 5 μg/mL G protein or BRSV. Supernatants from stimulated PBMCs and TBLN cells were collected and analyzed by ELISA for IFNγ **(A, C)** and IL-17 **(B, D)** secretion. Controls (n = 18), In + in BRSV-F/G CpG (n = 18), In + Sc BRSV-F/G CpG (n = 17), commercial vaccine (n = 11). Data represent means ± SEM. Statistical significance was determined by two-way ANOVA with Tukey’s *post hoc* test. *p<0.10 compared to matched mock controls.

Virus-specific IFNγ and IL-17 secretion was measured in cell culture supernatants of stimulated TBLN cells (**Figures 6A, B**) and PBMCs (**Figures 6C, D**) collected on 7 d.p.i. TBLN from calves that received In + Sc BRSV-F/G CpG produced more G-specific IFNγ (p ***<0.001) and IL-17 (*p=*0.054) secretion compared to their matched mock (**Figure 6A**). Also, TBLN cells from In + Sc BRSV-F/G CPG vaccinated animals tended to produce more G-specific IFNγ (p=0.098, **Figure 6A**) compared to unvaccinated control calves and more IL-17 (p=0.081, **Figure 6B**) compared to In + In BRSV-F/G CpG calves. TBLN cells from calves that received the MLV vaccine produced more IFNγ following restimulation with BRSV (*p=*0.054) and G protein (**p < 0.01) (**Figure 6A**) and more IL-17 in response to F protein (**p<0.01, **Figure 6B**) compared to the respective matched mock. TBLN cells from MLV immunized calves had higher BRSV-specific IFNγ responses compared to the calves that received the In + In BRSV-F/G CpG nanovaccine (*p=*0.082) (**Figure 6A**). The MLV vaccine also induced greater F-specific IL-17 responses in TBLN compared to the unvaccinated and In + In BRSV-F/G CpG calves (*p=*0.005 and *p=*0.0146 respectively, **Figure 6B**). No antigen-specific cytokine secretion was observed in TBLN cells from the unvaccinated control group or the In + In BRSV-F/G CpG in comparison to their matched mock (**Figures 6A, B**). TBLN cells from unvaccinated calves produced more G-specific IFNγ (**Figure 6A**) and IL-17A (**Figure 6B**) compared to the matched mocks, although this increase was numerical only and did not reach statistical significance (*p=*0.163 and *p=*0.141, respectively).

PBMCs from In + Sc nanovaccine calves produced more IFNγ in response to whole virus stimulation compared to their matched unstimulated mock cells (**p<0.01). We also observed greater BRSV-specific IFNγ responses in calves that received the In + Sc BRSV-F/G CpG nanovaccine and MLV vaccine compared to non-vaccinated control calves (*p=*0.020 and *p=*0.053 respectively) and compared to In + In BRSV-F/G CpG calves (*p=*0.017 and *p=*0.045 respectively) (**Figure 6C**). We observed no differences in PBMC antigen-specific IL-17 secretion amongst treatment groups. However, we noted G-specific IL-17 responses by PBMCs from both groups of BRSV-F/G CpG nanovaccine immunized groups compared to their matched mock (*p<0.05 for both) and in the unvaccinated group compared to its matched mock (p= 0.008) (**Figure 6D**).

### Principal component analysis for correlates of protection

**Figure 7:**
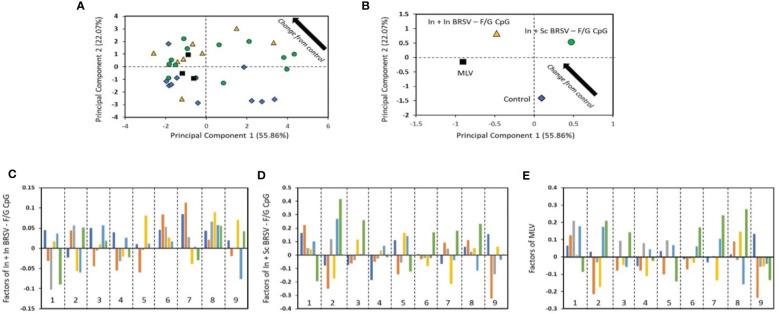
The results from the blinded PC analysis. **(A)** and **(B)** Scores plot from the analysis, with **(B)** showing the average treatment values. **(C–E)** show the impact of the treatment relative to control. The bars represent the disease protection descriptions and viral protection: total clinical score (dark blue), gross lung pathology (orange), lung histopathology score (gray), BAL cytology (yellow), nasal virus shedding (light blue) and lung viral burden (green). The numbered regions are the immune responses: (1) Serum neutralizing Ab titers, (2) nasal fluid IgA responses, (3) F specific IgG ELISA, (4) G specific IgG ELISA, (5) BRSV specific IgG ELISA, (6) cytokine responses by TBLN, (7) cytokine responses by PBMC, (8) TBLN proliferative responses, and (9) proliferative PBMC responses.

To investigate the underlying correlations of protection linked to the observed disease and viral protection of the vaccinated calves, PCA (62–65) was performed in a blinded manner on the immune parameters associated with both humoral and cellular responses, compared to the clinical and virological data of the vaccinated calves. Comparison of these immune responses between vaccinated calves and control calves by PCA was able to discriminate animals by vaccine groups (**Figure 7**). In order to perform this analysis, a new approach which first parameterizes the data and then performs a correlative analysis was developed (described in the methods section) (62–65). From the analysis, we were able to assess the differences in the treatments as compared to the control (**Figure 7A**). As the values decrease in principal component (PC) 1 and increase in PC2 (i.e., move to upper left in the PC1-PC2 plot), the treatment is more different than the control. From this analysis, we identified that animals receiving the In + Sc BRSV-F/G were the most different from the control group, followed by MLV, and finally In + In BRSV-F/G, indicating differential vaccine-elicited immune responses between vaccinated groups (**Figure 7B**). Additionally, the contributors to the differences between the treatments and the controls were identified through the analysis (**Figures 7C–E**). A larger bar indicates that the impact of the protection and immune responses are more different from the control unvaccinated calves. The sign of the bar (+ or -) is a comparative value, with the different signs indicating opposite effects. Increasing the values of features corresponding with large values positively differentiates from the control, while increasing features with negative values makes the treatment more like the control. Bars with little or no value are not impacted by the vaccine. For the most different vaccine group from control, the In + Sc BRSV-F/G (**Figure 7D**), we noted that serum neutralizing titers, nasal fluid IgA responses, and virus-specific PBMC proliferative responses have the largest effect from treatment, with the gross pathology lung score, nasal virus shedding and lung viral burden being the protection descriptions which are most changed. The largest difference with the other treatments is that In + In BRSV – F/G CpG shows larger impact on serum BRSV specific IgG ELISA and PMBC cytokines, as compared with In + Sc BRSV – F/G CpG, while MLV shows larger impact on lymph nodes cell and PBMC proliferation.

The analysis considers the relationships across all of the groups simultaneously while also providing a completely unbiased result. Some trends may not agree exactly with the observed results as PCA is identifying the main factor determining the trends and therefore focused on causality over correlation. The conclusion from PCA is that the main factors contributing to separation of the vaccinates from the controls are the: lung viral burden, which is impacted by nasal fluid IgA responses; lung histopathology score, which is most impacted by serum neutralizing antibody titers and nasal fluid IgA responses; and lung gross pathology score which is influenced by PBMC proliferative and cytokine responses. The factor that most differentiates the In + In BRSV – F/G CpG treatment from the other vaccinated groups is that the lung viral burden and lymph node proliferative responses are not negatively correlated with the control, as compared with the other treatments.

## Discussion

Previously, we developed a single dose, intranasal 50:50 CPTEG : CPH BRSV-Post-F/G nanovaccine that was successful in inducing a virus-specific IgA response and partial protection against BRSV challenge, but failed to induce significant circulating IgG responses in the face of high maternal antibody titers (27). Building on this work (27), the current study aimed to further optimize the BRSV-Post-F/G formulation by incorporating CpG ODN 2007 as an additional adjuvant, adding a prime/boost immunization regimen, and changing the polyanhydride NP chemistry from 50:50 CPTEG : CPH to 20:80 CPTEG : CPH. The magnitude and character of the immune response induced by the NPs can be influenced by changing the copolymer composition of the polyanhydride NPs. The change in ratio from 50:50 to the 20:80 CPTEG : CPH formulation was chosen to result in a more hydrophobic NP, with slower degradation and more sustained release kinetics of payloads (as observed in **Figure 1B**), an improved capacity to stabilize subunit proteins (as observed in **Figures 1C, D**), and an ability to induce long-lived immune responses (51, 54, 56, 66–71). Thus, we postulated that this change would induce more robust and sustained humoral and cell-mediated immune responses in our neonatal calf model. Indeed, in one study comparing the effect of polymer chemistries on the response to a model antigen, ovalbumin (OVA), the 20:80 CPTEG : CPH formulation was superior to the 50:50 CPTEG : CPH formulation, inducing a greater CD8^+^ T cell and serum antibody responses, and resulting in longer protection against an OVA-expressing tumor cell line (53). More recent studies by our group have shown similarly promising results with the 20:80 CPTEG : CPH-based nanovaccine formulation for inducing protective immunity against influenza (71, 72) and HRSV (73). To enhance the intrinsic immunostimulant properties of polyanhydride NPs, additional payloads such as TLR agonists can be incorporated as co-adjuvants. Incorporation of CpG, a potent TLR9 agonist, as an adjuvant has been used to improve the efficacy of other viral vaccines against influenza virus (71, 72) and HRSV (74, 75) by shaping the type of immune response to the pathogen. In work from our own group, CpG has also proven efficacious as a co-adjuvant in influenza (71, 72) and RSV (73). Therefore, we predicted that use of CpG would increase the vaccine’s immunogenicity and further improve the efficacy of the nanovaccine in neonates with MDA antibody.

In the present study, regardless of the prime-boost dosing regimen used, the BRSV-F/G CpG nanovaccine induced strong clinical, pathological, and virological protection in both the URT and LRT against BRSV infection and caused no vaccine enhanced disease in young calves. In comparison to our prior formulation (27, 55), we observed greater protection in calves receiving the optimized vaccine, including reduced viral burden in the LRT and less gross lung pathology (**Figures 2**, **4**). While the intranasal/intranasal nanovaccine regimen induced some protection, the heterologous prime/boost approach performed better in nearly all parameters of disease, including viral load in the LRT and induction of cellular immune responses (**Figures 4**, **6**, **7**). However, despite improving the immunogenicity and efficacy of the BRSV-F/G nanovaccine, we still failed to induce systemic humoral immune responses in calves with MDA, because we observed no significant differences between treatment groups in serum F-, G- or BRSV-specific IgG, or in serum neutralizing titers related to the vaccination or BRSV challenge (**Tables 1**, **2**). MDA are important during the first months of life in neonatal calves (76) and infants (77) to provide protection while the immune system fully matures. However, there are also numerous reports demonstrating that MDA can interfere with vaccine-induced responses in both calves (78) and infants (79), resulting in reduced or inadequate protection despite vaccination. Interestingly, there is growing evidence that antigen selection may be the most important factor for overcoming MDA. The pre-fusion F (preF) protein has been shown to be a critical target for neutralizing antibodies directed against both BRSV (24, 25, 80) and HRSV (21, 81). In a study by Riffault et al., immunization of 3–7 weeks of age calves with MDA with a single intramuscular injection of preF with Montanide™ ISA61^VG^ as an adjuvant conferred clinical and virological protection against BRSV infection (25). The vaccine elicited high virus neutralizing antibody titers following vaccination and infection and higher BRSV-specific circulating IgG and IgA antibodies titers compared to control calves (25). Interestingly, in humans, recent clinical trials have shown that vaccines based on the preF protein are highly efficacious in healthy adults (https://clinicaltrials.gov/ct2/show/NCT05035212, Pfizer), but studies with the preF in maternal vaccination studies have been less promising (https://clinicaltrials.gov/ct2/show/NCT02624947, Novavax). In recent work from our collaborative group, encapsulation of the HRSV preF antigen with CpG in a 20:80 CPTEG : CPH-based nanovaccine was highly efficacious in rodents (73, 82). Thus, future studies utilizing the nanovaccine platform combined with the preF alone or in combination with other RSV antigens such as the G protein may be the key to providing protection against BRSV infection in neonates with MDA.

There is paucity of clear correlates of protection for RSV in humans. It is unclear whether systemic or local IgG, local mucosal IgA, cellular immunity, or a combination of these responses are required for an efficacious RSV vaccine (83, 84). There are, however, multiple reports indicating that IgA plays a critical role in protection from RSV infection (85–88), while both systemic and mucosal IgG may have a more important role in long-term resistance and reinfection (89). The success of prophylactic use of neutralizing mAb therapeutics in humans (23, 90) and additional results from a recent meta-analyses study integrating humoral and cellular immune responses data suggest that serum neutralizing antibodies are most important in reducing disease (91). Although, this is somewhat belied by the evidence that infants and young calves with high MDA are still susceptible to severe RSV disease (25). In the report by Riffault et al., a PCA analysis revealed that neither total BRSV-specific, preF-specific IgA, nor IFNγ-producing T cell responses correlated with virological protection in calves with MDA, but that serum preF titers were the most important factor associated with protection (25). Recent evidence in non-human primates suggests that correlates of protection against RSV differ by compartment (92). Following an experimental RSV challenge, virus-specific IgA titers, neutralization antibodies, and complement activity were corelated to URT protection while LRT protection was associated with Fc-mediated antibody effector activity, supporting the importance of non-neutralizing-antibody functions in RSV protection (92). It has been demonstrated that the use of simultaneous and/or sequential mucosal and parenteral vaccination induces both local mucosal IgA and systemic IgG and cell mediated responses in other disease models (73, 93, 94). In the present study, the magnitude of mucosal and cellular responses differed between subcutaneous and intranasal boost immunization strategies. Intranasal priming followed by subcutaneous boosting induced mucosal IgA in the BAL samples from Study #1 (n=6) while homologous intranasal prime/boost dosing did not (**Table 3**). In our previous work, we observed robust virus-specific IgA responses after a single intranasal immunization with the 50:50 CPTEG : CPH nanovaccine formulation (27, 55). Due to technical difficulties in obtaining BAL samples from Study #2, it was not possible to evaluate BAL IgA in all calves. In our trial, subcutaneous boosting but not intranasal boosting with the BRSV-Post-F/G nanovaccine induced cellular responses characterized by secretion of virus-specific IL-17 and IFNγ (**Figure 6**).

PCA analysis of the immune, virological, and clinical data of the vaccinated calves identified that no single parameter directly correlated with protection, but a combination of circulating and mucosal humoral and cellular responses were important common correlates of immunity in the pathological and clinical protection induced by the vaccination. In calves receiving a single shot of preF vaccine, PCA analysis revealed that neutralizing antibodies, BRSV specific serum IgG antibody responses, and antibodies to preF were significant correlates of protection (25, 95). In the present study, even though our current formulations did not induce significantly strong BRSV serum neutralization titers or mucosal IgA responses, PCA analysis shows that they corresponded to viral control in the LRT, while the most important parameters associated with less severe pathology were nasal fluid IgA and cellular responses (**Figure 7**). Together, our results suggest that the use of the heterologous prime/boost immunization regimen may be the ideal approach to induce an appropriate combination of cellular and humoral immune responses.

To the best of our knowledge, this is the first study to compare the response to a subunit-based nanovaccine and a licensed MLV vaccine against any animal disease, including BRSV (96). Although our study design was not powered adequately to differentiate between the efficacy of the two vaccine approaches, our results suggest that the intranasal/subcutaneous BRSV-F/G CpG vaccine performed at least as well as the MLV in terms of disease and virological protection against BRSV challenge. MLV vaccines are cheap to develop but often require multiple booster immunizations (97–99) and pose safety concerns due to shedding of the vaccine virus by vaccinated calves and potentially spreading them to naïve animals (100). Moreover, immunization with an MLV during an active course of natural BRSV infection in a herd can result in vaccine enhanced disease, which has been previously reported (101). In humans, CDC recommends against administering live attenuated virus vaccines to infants and people who are severely immunocompromised or pregnant, the latter due to the theoretical risk to the fetus (https://www.cdc.gov/vaccines/hcp/acip-recs/general-recs/contraindications.html). Thus, a subunit vaccine is safer for high-risk populations. The depot effect offered by NPs is appealing, because the long-term release of antigen by the surface eroding NPs has the capacity to induce more sustained immune responses without the need for a booster vaccine. Polyanhydride nanovaccines have been shown to enhance germinal center B cell formation (48) and are known to elicit sustained high avidity serum antibody responses to a broad range of pathogens (51, 54, 63, 66, 67, 69–71, 102). While in the current study, we opted for a two-dose approach due to challenges associated with the neonatal immune system and competing MDAs, results from other nanovaccine studies suggest that with the optimal formulation and antigen combination(s), a single dose may be sufficient to induce long-term protection from infection (25, 54, 95). Thus, our flexible and plug-n-play NP-based design is safer and allows for tunable antigen release kinetics and diverse antigen loading strategies. Altogether, these attributes make polyanhydride-based NP platforms a strong prospect for next-generation vaccine development against both human and animal disease (47, 103).

## Data availability statement

The original contributions presented in the study are included in the article/supplementary material. Further inquiries can be directed to the corresponding author.

## Ethics statement

The animal study was reviewed and approved by Iowa State University Institutional Animal Care and Use Committee.

## Author contributions

Conceptualization, JM, BN, RS. performed the experiments and analyzed the data, TM, EG, SB, manuscript writing, TM, EG, SB, RS, BN, JM. All authors contributed to the article and approved the submitted version.

## References

[1] Nair H Nokes DJ Gessner BD Dherani M Madhi SA Singleton RJ. Global burden of acute lower respiratory infections due to respiratory syncytial virus in young children: a systematic review and meta-analysis. Lancet (2010) 375:1545–55. doi: 10.1016/S0140-6736(10)60206-1 PMC2864404 20399493

[2] Shi T Denouel A Tietjen AK Campbell I Moran E Li X. Global disease burden estimates of respiratory syncytial virus-associated acute respiratory infection in older adults in 2015: a systematic review and meta-analysis. J Infect Dis (2020) 222:S577–83. doi: 10.1093/infdis/jiz059 30880339

[3] Shi T McAllister DA O'Brien KL Simoes EAF Madhi SA Gessner GD. Global, regional, and national disease burden estimates of acute lower respiratory infections due to respiratory syncytial virus in young children in 2015: a systematic review and modelling study. Lancet (2017) 390:946–58. doi: 10.1016/S0140-6736(17)30938-8 PMC5592248 28689664

[4] Hall CB Walsh EE Long CE Schnabel KC. Immunity to and frequency of reinfection with respiratory syncytial virus. J Infect Dis (1991) 163:693–8. doi: 10.1093/infdis/163.4.693 2010624

[5] Henderson FW Collier AM Clyde WA Denny FW. Respiratory-syncytial-virus infections, reinfections and immunity. a prospective, longitudinal study in young children. N Engl J Med (1979) 300:530–4. doi: 10.1056/NEJM197903083001004 763253

[6] Bont L Versteegh J Swelsen WTN Heijnen CJ Kavelaars A Brus F. Natural reinfection with respiratory syncytial virus does not boost virus-specific T-cell immunity. Pediatr Res (2002) 52:363–7. doi: 10.1203/00006450-200209000-00009 12193668

[7] Bozzola E Barni S Villani A. Respiratory syncytial virus pediatric hospitalization in the COVID-19 era. Int J Environ Res Public Health (2022) 19:15455. doi: 10.3390/ijerph192315455 36497528 PMC9738890

[8] De Rose DU Caoci S Auriti C Maddaloni C Capolupo I Salvatori G. Lessons from SARS-CoV-2 pandemics: how restrictive measures impacted the trend of respiratory infections in neonates and infants up to three months of age. Pathogens (2022) 11:1086. doi: 10.3390/pathogens11101086 36297143 PMC9611509

[9] Altamirano-Lagos MJ Diaz FE Mansilla MA Rivera-Perez D Soto D McGill JL. Current animal models for understanding the pathology caused by the respiratory syncytial virus. Front Microbiol (2019) 10:873. doi: 10.3389/fmicb.2019.00873 31130923 PMC6510261

[10] Stokes KL Chi MH Sakamoto K Newcomb DC Currier MG Huckabee MM. Differential pathogenesis of respiratory syncytial virus clinical isolates in BALB/c mice. J Virol (2011) 85:5782–93. doi: 10.1128/JVI.01693-10 PMC3126300 21471228

[11] Guerra-Maupome M Palmer MV McGill JL Sacco RE. Utility of the neonatal calf model for testing vaccines and intervention strategies for use against human RSV infection. Vaccines (Basel) (2019) 7:7. doi: 10.3390/vaccines7010007 30626099 PMC6466205

[12] Booker CW Abutarbush SM Morley PS Jim GK Pittman TJ Schunight OC. Microbiological and histopathological findings in cases of fatal bovine respiratory disease of feedlot cattle in Western Canada. Can Vet J (2008) 49:473–81. PMC2359492 18512458

[13] Van der Poel WH Brand A Kramps JA Van Oirschot JT. Respiratory syncytial virus infections in human beings and in cattle. J Infect (1994) 29:215–28. doi: 10.1016/s0163-4453(94)90866-4 7806887

[14] Sacco RE McGill JL Palmer MV Lippolis JD Reinhardt TA Nonnecke BJ. Neonatal calf infection with respiratory syncytial virus: drawing parallels to the disease in human infants. Viruses (2012) 4:3731–53. doi: 10.3390/v4123731 PMC3528288 23342375

[15] McLellan JS Yang Y Graham BS Kwong PD. Structure of respiratory syncytial virus fusion glycoprotein in the postfusion conformation reveals preservation of neutralizing epitopes. J Virol (2011) 85:7788–96. doi: 10.1128/JVI.00555-11 PMC3147929 21613394

[16] McLellan JS Chen M Leung S Graepel KW Du X Yang Y. Structure of RSV fusion glycoprotein trimer bound to a prefusion-specific neutralizing antibody. Science (2013) 340:1113–7. doi: 10.1126/science.1234914 PMC4459498 23618766

[17] McLellan JS. Neutralizing epitopes on the respiratory syncytial virus fusion glycoprotein. Curr Opin Virol (2015) 11:70–5. doi: 10.1016/j.coviro.2015.03.002 PMC4456247 25819327

[18] Swanson KA Settembre EC Shaw CA Dey AK Rappouli R Mandl CW. Structural basis for immunization with postfusion respiratory syncytial virus fusion f glycoprotein (RSV f) to elicit high neutralizing antibody titers. Proc Natl Acad Sci USA (2011) 108:9619–24. doi: 10.1073/pnas.1106536108 PMC3111287 21586636

[19] Raghunandan R Lu H Zhou B Xabier MG Massare MJ Flyer DC. An insect cell derived respiratory syncytial virus (RSV) f nanoparticle vaccine induces antigenic site II antibodies and protects against RSV challenge in cotton rats by active and passive immunization. Vaccine (2014) 32:6485–92. doi: 10.1016/j.vaccine.2014.09.030 PMC7172787 25269094

[20] McGinnes Cullen L Schmidt MR Kenward SA Woodland RT Morrison TG. Murine immune responses to virus-like particle-associated pre- and postfusion forms of the respiratory syncytial virus f protein. J Virol (2015) 89:6835–47. doi: 10.1128/JVI.00384-15 PMC4468467 25903340

[21] Capella C Prefusion F Postfusion F Antibodies G. And disease severity in infants and young children with acute respiratory syncytial virus infection. J Infect Dis (2017) 216:1398–406. doi: 10.1093/infdis/jix489 PMC5853469 29029312

[22] Sadoff J De Paepe E DeVincenzo J Gymnopoulou E Menten J Murray B. Prevention of respiratory syncytial virus infection in healthy adults by a single immunization of Ad26.RSV.preF in a human challenge study. J Infect Dis (2021) 226:396–406. doi: 10.1093/infdis/jiab003 PMC9417128 33400792

[23] Magro M Mas V Chappell K Vazquez M Cano O Luque D. Neutralizing antibodies against the preactive form of respiratory syncytial virus fusion protein offer unique possibilities for clinical intervention. Proc Natl Acad Sci USA (2012) 109:3089–94. doi: 10.1073/pnas.1115941109 PMC3286924 22323598

[24] Zhang B Chen L Silacci C Thom M Boyington JC Druz A. Protection of calves by a prefusion-stabilized bovine RSV f vaccine. NPJ Vaccines (2017) 2:7. doi: 10.1038/s41541-017-0005-9 29021918 PMC5627276

[25] Riffault S Hagglund Guzman S E Naslund K Jouneau L Dubuquoy C. A single shot pre-fusion-Stabilized bovine RSV f vaccine is safe and effective in newborn calves with maternally derived antibodies. Vaccines (Basel) (2020) 8:231. doi: 10.3390/vaccines8020231 32443437 PMC7349975

[26] Riffault S Meyer G Deplanche M Dubuquoy C Durand G Soulestin M. A new subunit vaccine based on nucleoprotein nanoparticles confers partial clinical and virological protection in calves against bovine respiratory syncytial virus. Vaccine (2010) 28:3722–34. doi: 10.1016/j.vaccine.2010.03.008 PMC7115569 20307593

[27] McGill JL Kelly S Kumar P Speckhart S Haughney SL Henningson J. Efficacy of mucosal polyanhydride nanovaccine against respiratory syncytial virus infection in the neonatal calf. Sci Rep (2018) 8:3021. doi: 10.1038/s41598-018-21292-2 29445124 PMC5813012

[28] Karger A Schmidt U Buchholz UJ. Recombinant bovine respiratory syncytial virus with deletions of the G or SH genes: G and f proteins bind heparin. J Gen Virol (2001) 82:631–40. doi: 10.1099/0022-1317-82-3-631 11172105

[29] Johnson PR Spriggs MK Olmsted RA Collins PL. The G glycoprotein of human respiratory syncytial viruses of subgroups a and b: extensive sequence divergence between antigenically related proteins. Proc Natl Acad Sci USA (1987) 84:5625–9. doi: 10.1073/pnas.84.16.5625 PMC298915 2441388

[30] Langedijk JP Meloen RH Taylor G Furze JM van Oirschot JT. Antigenic structure of the central conserved region of protein G of bovine respiratory syncytial virus. J Virol (1997) 71:4055–61. doi: 10.1128/JVI.71.5.4055-4061.1997 PMC191558 9094683

[31] Langedijk JP Schaaper WM Meloen RH van Oirschot JT. Proposed three-dimensional model for the attachment protein G of respiratory syncytial virus. J Gen Virol (1996) 77(Pt 6):1249–57. doi: 10.1099/0022-1317-77-6-1249 8683213

[32] Fedechkin SO George NL Castrejon AMN Dillen JR Kauvar LM DuBois RM. Conformational flexibility in respiratory syncytial virus G neutralizing epitopes. J Virol (2020) 94:e01879–19. doi: 10.1128/JVI.01879-19 PMC7158734 31852779

[33] Fuentes S Klenow L Golding H Khurana S. Preclinical evaluation of bacterially produced RSV-G protein vaccine: strong protection against RSV challenge in cotton rat model. Sci Rep (2017) 7:42428. doi: 10.1038/srep42428 28186208 PMC5301242

[34] Haynes LM Caidi H Radu GU Miao C Harcourt JL Tripp RA. Therapeutic monoclonal antibody treatment targeting respiratory syncytial virus (RSV) G protein mediates viral clearance and reduces the pathogenesis of RSV infection in BALB/c mice. J Infect Dis (2009) 200:439–47. doi: 10.1086/600108 19545210

[35] Lee HJ Lee JY Park MH Kim JY Chang J. Monoclonal antibody against G glycoprotein increases respiratory syncytial virus clearance *In vivo* and prevents vaccine-enhanced diseases. PloS One (2017) 12:e0169139. doi: 10.1371/journal.pone.0169139 28076422 PMC5226777

[36] Fuentes S Coyle EM Golding H Khurana S. Nonglycosylated G-protein vaccine protects against homologous and heterologous respiratory syncytial virus (RSV) challenge, while glycosylated G enhances RSV lung pathology and cytokine levels. J Virol (2015) 89:8193–205. doi: 10.1128/JVI.00133-15 PMC4524247 26018164

[37] Lee J Klenow L Coyle EM Golding H Khurana S. Protective antigenic sites in respiratory syncytial virus G attachment protein outside the central conserved and cysteine noose domains. PloS Pathog (2018) 14:e1007262. doi: 10.1371/journal.ppat.1007262 30142227 PMC6126872

[38] Nuñez Castrejon AM O'Rourke SM Kauvar LM DuBois RM. Structure-based design and antigenic validation of respiratory syncytial virus G immunogens. J Virol (2022) 96:e0220121. doi: 10.1128/jvi.02201-21 35266806 PMC9006937

[39] Tripp RA Power UF Openshaw PJM Kauvar LM. Respiratory syncytial virus: targeting the G protein provides a new approach for an old problem. J Virol (2018) 92:e01302–17. doi: 10.1128/JVI.01302-17 PMC5774885 29118126

[40] Schrijver RS Langedijk JPM Keil GM Middel WGJ Maris-Veldhuis M Oirschot JTV. Immunization of cattle with a BHV1 vector vaccine or a DNA vaccine both coding for the G protein of BRSV. Vaccine (1997) 15:1908–16. doi: 10.1016/s0264-410x(97)00129-1 9413101

[41] Bastien N Taylor G Thomas LH Wyld SG Simard C Trudel M. Immunization with a peptide derived from the G glycoprotein of bovine respiratory syncytial virus (BRSV) reduces the incidence of BRSV-associated pneumonia in the natural host. Vaccine (1997) 15:1385–90. doi: 10.1016/s0264-410x(97)00033-9 9302749

[42] Taylor G Thomas LH Furze JM Cook RS Wyld SG Lerch R. Recombinant vaccinia viruses expressing the f, G or n, but not the M2, protein of bovine respiratory syncytial virus (BRSV) induce resistance to BRSV challenge in the calf and protect against the development of pneumonic lesions. J Gen Virol (1997) 78(Pt 12):3195–206. doi: 10.1099/0022-1317-78-12-3195 9400970

[43] Fuentes S Hahn M Chilcote K Chemaly RF Shah DP Ye X. Antigenic fingerprinting of respiratory syncytial virus (RSV)-A-Infected hematopoietic cell transplant recipients reveals importance of mucosal anti-RSV G antibodies in control of RSV infection in humans. J Infect Dis (2020) 221:636–46. doi: 10.1093/infdis/jiz608 PMC7530544 31745552

[44] Mazur NI Lowensteyn YN Willemsen JE Gill CJ Forman L Mwananyanda LM. Global respiratory syncytial virus-related infant community deaths. Clin Infect Dis (2021) 73:S229–37. doi: 10.1093/cid/ciab528 PMC8411255 34472576

[45] Grego EA Siddoway AC Uz M Liu L Christansen JC Ross KA. Polymeric nanoparticle-based vaccine adjuvants and delivery vehicles. Curr Top Microbiol Immunol (2021) 433:29–76. doi: 10.1007/82_2020_226 33165869 PMC8107186

[46] Wilson-Welder JH Torrse MP Kipper MJ Mallapragada SK Wannemuehler MJ Narasimhan B. Vaccine adjuvants: current challenges and future approaches. J Pharm Sci (2009) 98:1278–316. doi: 10.1002/jps.21523 PMC8092333 18704954

[47] Maina TW Grego EA Boggiatto PM Sacco RE Narasimhan B McGill JL. Applications of nanovaccines for disease prevention in cattle. Front Bioeng Biotechnol (2020) 8:608050. doi: 10.3389/fbioe.2020.608050 33363134 PMC7759628

[48] Vela Ramirez JE Tygrett LT Hao J Habte HH Cho MW Greenspan NS. Polyanhydride nanovaccines induce germinal center b cell formation and sustained serum antibody responses. J BioMed Nanotechnol (2016) 12:1303–11. doi: 10.1166/jbn.2016.2242 PMC5438750 27319223

[49] Vela-Ramirez JE Goodman JT Boggiatto PM Roychoudhury R Pohl NLB Hostetter JM. Safety and biocompatibility of carbohydrate-functionalized polyanhydride nanoparticles. AAPS J (2015) 17:256–67. doi: 10.1208/s12248-014-9699-z PMC4287302 25421457

[50] Vela Ramirez JE Roychoudhury R Habte HH Cho MW Pohl NLB Narasimhan B. Carbohydrate-functionalized nanovaccines preserve HIV-1 antigen stability and activate antigen presenting cells. J Biomater Sci Polym Ed (2014) 25:1387–406. doi: 10.1080/09205063.2014.940243 PMC4465578 25068589

[51] Huntimer L Wilson-Welder JH Ross K Carrillo-Conde B Pruisner L Wang C. Single immunization with a suboptimal antigen dose encapsulated into polyanhydride microparticles promotes high titer and avid antibody responses. J BioMed Mater Res B Appl Biomater (2013) 101:91–8. doi: 10.1002/jbm.b.32820 23143744

[52] Lopac SK Torres MP Wilson-Welder JH Wannemuehler MJ Narasimhan B. Effect of polymer chemistry and fabrication method on protein release and stability from polyanhydride microspheres. J BioMed Mater Res B Appl Biomater (2009) 91:938–47. doi: 10.1002/jbm.b.31478 PMC3710783 19642209

[53] Wafa EI Geary SM Goodman JT Narasimhan B Salem AK. The effect of polyanhydride chemistry in particle-based cancer vaccines on the magnitude of the anti-tumor immune response. Acta Biomater (2017) 50:417–27. doi: 10.1016/j.actbio.2017.01.005 PMC5316298 28063991

[54] Ulery BD Kumar D Ramer-Tait AE Metzger DW Wannemuehler MJ Narasimhan B. Design of a protective single-dose intranasal nanoparticle-based vaccine platform for respiratory infectious diseases. PloS One (2011) 6:e17642. doi: 10.1371/journal.pone.0017642 21408610 PMC3048296

[55] McGill JL Kelly SM Guerra-Maupone M Winkley E Henningson J Narasimhan B. Vitamin a deficiency impairs the immune response to intranasal vaccination and RSV infection in neonatal calves. Sci Rep (2019) 9:15157. doi: 10.1038/s41598-019-51684-x 31641172 PMC6805856

[56] Torres MP Determan AS Anderson GL Mallapragada SK Narasimhan B. Amphiphilic polyanhydrides for protein stabilization and release. Biomaterials (2007) 28:108–16. doi: 10.1016/j.biomaterials.2006.08.047 PMC8100984 16965812

[57] Ulery BD Phanse Y Sinha A Wannemuehler MJ Narasimhan B Bellaire BH. Polymer chemistry influences monocytic uptake of polyanhydride nanospheres. Pharm Res (2009) 26:683–90. doi: 10.1007/s11095-008-9760-7 18987960

[58] Wagner DA Kelly SM Petersen AC Peroutka-Bigus N Darling RJ Bellaire BH. Single-dose combination nanovaccine induces both rapid and long-lived protection against pneumonic plague. Acta Biomater (2019) 100:326–37. doi: 10.1016/j.actbio.2019.10.016 PMC7012387 31610342

[59] Díaz FE Guerra-Maupome M McDonald PO Rivera-Perez D Kalergis AM McGill JL. A recombinant BCG vaccine is safe and immunogenic in neonatal calves and reduces the clinical disease caused by the respiratory syncytial virus. Front Immunol (2021) 12:664212. doi: 10.3389/fimmu.2021.664212 33981309 PMC8108697

[60] Wagner-Muñiz DA Haughney SL Kelly SM Wannemuehler MJ Narasimhan B. Room temperature stable PspA-based nanovaccine induces protective immunity. Front Immunol (2018) 9:325. doi: 10.3389/fimmu.2018.00325 29599766 PMC5863507

[61] Sacco RE McGill JL Pillatzki AE Palmer MV Ackermann MR. Respiratory syncytial virus infection in cattle. Vet Pathol (2014) 51:427–36. doi: 10.1177/0300985813501341 24009269

[62] Jolliffe IT Cadima J. Principal component analysis: a review and recent developments. Philos Trans A Math Phys Eng Sci (2016) 374:20150202. doi: 10.1098/rsta.2015.0202 26953178 PMC4792409

[63] Ross KA Adams J Loyd H Ahmed S Samboli A Broderick S. Combination nanovaccine demonstrates synergistic enhancement in efficacy against influenza. ACS Biomater Sci Eng (2016) 2:368–74. doi: 10.1021/acsbiomaterials.5b00477 33429541

[64] Phanse Y Puttamreddy S Loy D Vela Ramirez J Ross KA Alvarez-Castro I. RNA Nanovaccine protects against white spot syndrome virus in shrimp. Vaccines (Basel) (2022) 10:1428. doi: 10.3390/vaccines10091428 36146509 PMC9504209

[65] Mullis AS Broderick SR Binnebose AM Peroutka-Bigus N Bellaire BH Rajan K. Data analytics approach for rational design of nanomedicines with programmable drug release. Mol Pharm (2019) 16:1917–28. doi: 10.1021/acs.molpharmaceut.8b01272 30973741

[66] Haughney SL Petersen LK Schoofs AD Ramer-Tait AE King JD Briles DE. Retention of structure, antigenicity, and biological function of pneumococcal surface protein a (PspA) released from polyanhydride nanoparticles. Acta Biomater (2013) 9:8262–71. doi: 10.1016/j.actbio.2013.06.006 PMC3777629 23774257

[67] Carrillo-Conde B Schiltz E Yu J Minion EC Phillips GJ Wannemuehler MJ. Encapsulation into amphiphilic polyanhydride microparticles stabilizes yersinia pestis antigens. Acta Biomater (2010) 6:3110–9. doi: 10.1016/j.actbio.2010.01.040 20123135

[68] Petersen LK Phanse Y Ramer-Tait AE Wannemuehler MJ Narasimhan B. Amphiphilic polyanhydride nanoparticles stabilize bacillus anthracis protective antigen. Mol Pharm (2012) 9:874–82. doi: 10.1021/mp2004059 PMC3325798 22380593

[69] Determan AS Wilson JH Kipper MJ Wannemuehler MJ Narasimhan B. Protein stability in the presence of polymer degradation products: consequences for controlled release formulations. Biomaterials (2006) 27:3312–20. doi: 10.1016/j.biomaterials.2006.01.054 16504288

[70] Haughney SL Ross KA Boggiatto PM Wannemuehler MJ Narasimhan B. Effect of nanovaccine chemistry on humoral immune response kinetics and maturation. Nanoscale (2014) 6:13770–8. doi: 10.1039/c4nr03724c 25285425

[71] Zacharias ZR Ross KA Hornick EE Goodman JT Narasimhan B Waldshmidt TJ. Polyanhydride nanovaccine induces robust pulmonary b and T cell immunity and confers protection against homologous and heterologous influenza a virus infections. Front Immunol (2018) 9:1953. doi: 10.3389/fimmu.2018.01953 30233573 PMC6127617

[72] Dhakal S Ghimire S Renu S Ross KA Lakshmanappa YS Hogshead BT. Evaluation of CpG-ODN-adjuvanted polyanhydride-based intranasal influenza nanovaccine in pigs. Vet Microbiol (2019) 237:108401. doi: 10.1016/j.vetmic.2019.108401 31585639

[73] Stephens LM Ross KA Waldstein KA Legge KL McLellan JS Narasimhan B. Prefusion f-based polyanhydride nanovaccine induces both humoral and cell-mediated immunity resulting in long-lasting protection against respiratory syncytial virus. J Immunol (2021) 206:2122–34. doi: 10.4049/jimmunol.2100018 PMC8062305 33827894

[74] Garlapati S Garg R Brownlie R Latimer L Simko E Hancock REW. Enhanced immune responses and protection by vaccination with respiratory syncytial virus fusion protein formulated with CpG oligodeoxynucleotide and innate defense regulator peptide in polyphosphazene microparticles. Vaccine (2012) 30:5206–14. doi: 10.1016/j.vaccine.2012.06.011 22713718

[75] Ma Y Jiao YY Yu YZ Jiang N Hua Y Zhang XJ. A built-in CpG adjuvant in RSV f protein DNA vaccine drives a Th1 polarized and enhanced protective immune response. Viruses (2018) 10:38. doi: 10.3390/v10010038 29342954 PMC5795451

[76] Chase CC Hurley DJ. And reber, A.J. neonatal immune development in the calf and its impact on vaccine response. Vet Clin North Am Food Anim Pract (2008) 24(1):87–104. doi: 10.1016/j.cvfa.2007.11.001 18299033 PMC7127081

[77] Niewiesk S. Maternal antibodies: clinical significance, mechanism of interference with immune responses, and possible vaccination strategies. Front Immunol (2014) 5:446. doi: 10.3389/fimmu.2014.00446 25278941 PMC4165321

[78] Windeyer MC Gamsjäger L. Vaccinating calves in the face of maternal antibodies: challenges and opportunities. Vet Clin North Am Food Anim Pract (2019) 35(3):557–73. doi: 10.1016/j.cvfa.2019.07.004 31590902

[79] Zimmermann P Jones CE. Factors that influence infant immunity and vaccine responses. Pediatr Infect Dis J (2021) 40(5S):S40–6. doi: 10.1097/INF.0000000000002773 34042910

[80] Steff AM Monroe J Fridrich K Chandramouli S Nguyen TLA Tian S. Pre-fusion RSV f strongly boosts pre-fusion specific neutralizing responses in cattle pre-exposed to bovine RSV. Nat Commun (2017) 8:1085. doi: 10.1038/s41467-017-01092-4 29057917 PMC5651886

[81] Ngwuta JO Chen M Modjarrad K Joyce MG Kenekiyo M Kumar A. Prefusion f-specific antibodies determine the magnitude of RSV neutralizing activity in human sera. Sci Transl Med (2015) 7:309ra162. doi: 10.1126/scitranslmed.aac4241 PMC4672383 26468324

[82] Stephens LM Ross KA McLellan JS Narasimhan B Varga SM. Long-lasting protection induced by a polyanhydride nanovaccine against respiratory syncytial virus in an outbred mouse model. J Virol (2022) 96:e0150222. doi: 10.1128/jvi.01502-22 36314826 PMC9683007

[83] Soto JA Stephens LM Waldstein KA Canedo-Marroquin G Varga SM Kalergis AM. Current insights in the development of efficacious vaccines against RSV. Front Immunol (2020) 11:1507. doi: 10.3389/fimmu.2020.01507 32765520 PMC7379152

[84] Openshaw PJM Chiu C Culley FJ Johansson C. Protective and harmful immunity to RSV infection. Annu Rev Immunol (2017) 35:501–32. doi: 10.1146/annurev-immunol-051116-052206 28226227

[85] Habibi MS Jozwik A Makris S Dunning J Paras A DeVincenzo JP. Impaired antibody-mediated protection and defective IgA b-cell memory in experimental infection of adults with respiratory syncytial virus. Am J Respir Crit Care Med (2015) 191:1040–9. doi: 10.1164/rccm.201412-2256OC PMC4435460 25730467

[86] Hijano DR Siefker DT Shrestha B Jaligama S Vu LD Tillman H. Type I interferon potentiates IgA immunity to respiratory syncytial virus infection during infancy. Sci Rep (2018) 8:11034. doi: 10.1038/s41598-018-29456-w 30038294 PMC6056463

[87] Reed JL Welliver TP Sims GP McKinney L Velozo L Avendano L. Innate immune signals modulate antiviral and polyreactive antibody responses during severe respiratory syncytial virus infection. J Infect Dis (2009) 199:1128–38. doi: 10.1086/597386 19278337

[88] Bagga B Cehelsky JE Vaishna A Wilkinson T Meyers R Harrison LM. Effect of preexisting serum and mucosal antibody on experimental respiratory syncytial virus (RSV) challenge and infection of adults. J Infect Dis (2015) 212:1719–25. doi: 10.1093/infdis/jiv281 25977264

[89] Vissers M Ahout IM de Jonge MI Ferwerda G. Mucosal IgG levels correlate better with respiratory syncytial virus load and inflammation than plasma IgG levels. Clin Vaccine Immunol (2015) 23:243–5. doi: 10.1128/CVI.00590-15 PMC4783421 26656116

[90] Robbie GJ Zhao L Mondick J Losonsky G Roskos LK. Population pharmacokinetics of palivizumab, a humanized anti-respiratory syncytial virus monoclonal antibody, in adults and children. Antimicrob Agents Chemother (2012) 56:4927–36. doi: 10.1128/AAC.06446-11 PMC3421858 22802243

[91] Maas BM Lommerse J Plock N Railkar RA Cheung SYA Caro L. Forward and reverse translational approaches to predict efficacy of neutralizing respiratory syncytial virus (RSV) antibody prophylaxis. EBioMedicine (2021) 73:103651. doi: 10.1016/j.ebiom.2021.103651 34775220 PMC8603022

[92] Zohar T Hsiao JC Mehta N Das J Devadhasan A Karpinski W. Upper and lower respiratory tract correlates of protection against respiratory syncytial virus following vaccination of nonhuman primates. Cell Host Microbe (2022) 30:41–52.e45. doi: 10.1016/j.chom.2021.11.006 34879230

[93] McCluskie MJ Weeratna RD Payette PJ Davis HL. Parenteral and mucosal prime-boost immunization strategies in mice with hepatitis b surface antigen and CpG DNA. FEMS Immunol Med Microbiol (2002) 32:179–85. doi: 10.1111/j.1574-695X.2002.tb00551.x 11934561

[94] Lapuente D Fuchs J Willar J Antao AV Eberlein V Uhlig N. Protective mucosal immunity against SARS-CoV-2 after heterologous systemic prime-mucosal boost immunization. Nat Commun (2021) 12:6871. doi: 10.1038/s41467-021-27063-4 34836955 PMC8626513

[95] Valarcher JF Hagglund S Naslund K Jouneau L Malmstrom E Boulesteix O. Single-shot vaccines against bovine respiratory syncytial virus (BRSV): comparative evaluation of long-term protection after immunization in the presence of BRSV-specific maternal antibodies. Vaccines (Basel) (2021) 9:236. doi: 10.3390/vaccines9030236 33803302 PMC8001206

[96] Martinez DA Newcomer B Passler T Chamorro MF. Efficacy of bovine respiratory syncytial virus vaccines to reduce morbidity and mortality in calves within experimental infection models: a systematic review and meta-analysis. Front Vet Sci (2022) 9:906636. doi: 10.3389/fvets.2022.906636 35782561 PMC9245045

[97] Palomares RA Bittar JHJ Woolums AR Hoyos-Jaramillo A Hurley DJ Saliki JT. Comparison of the immune response following subcutaneous versus intranasal modified-live virus booster vaccination against bovine respiratory disease in pre-weaning beef calves that had received primary vaccination by the intranasal route. Vet Immunol Immunopathol (2021) 237:110254. doi: 10.1016/j.vetimm.2021.110254 34034143

[98] Woolums AR Berghaus RD Berhaus LJ Ellis RW Pence ME Saliki JT. Effect of calf age and administration route of initial multivalent modified-live virus vaccine on humoral and cell-mediated immune responses following subsequent administration of a booster vaccination at weaning in beef calves. Am J Vet Res (2013) 74:343–54. doi: 10.2460/ajvr.74.2.343 23363363

[99] Ellis J Gow S Berenik A Lacoste S Erickson N. Comparative efficacy of modified-live and inactivated vaccines in boosting responses to bovine respiratory syncytial virus following neonatal mucosal priming of beef calves. Can Vet J (2018) 59:1311–9. PMC6237255 30532289

[100] Kim HW Arrobio JO Brandt CD Wright P Hodes D Chanock RM. Safety and antigenicity of temperature sensitive (TS) mutant respiratory syncytial virus (RSV) in infants and children. Pediatrics (1973) 52:56–63. doi: 10.1542/peds.52.1.56 4353352

[101] Kimman TG Sol J Westenbrink F Straver PJ. A severe outbreak of respiratory tract disease associated with bovine respiratory syncytial virus probably enhanced by vaccination with modified live vaccine. Vet Q (1989) 11:250–3. doi: 10.1080/01652176.1989.9694231 2603358

[102] Huntimer L Ramer-Tait AE Petersen LK Ross KA Walz KA Wang C. Evaluation of biocompatibility and administration site reactogenicity of polyanhydride-particle-based platform for vaccine delivery. Adv Healthc Mater (2013) 2:369–78. doi: 10.1002/adhm.201200181 23184561

[103] Aida V Pliasas VC Neasham PJ North JF McWhorter KL Glover SR. Novel vaccine technologies in veterinary medicine: a herald to human medicine vaccines. Front Vet Sci (2021) 8:654289. doi: 10.3389/fvets.2021.654289 33937377 PMC8083957

